# Prediction of plant pre-microRNAs and their microRNAs in genome-scale sequences using structure-sequence features and support vector machine

**DOI:** 10.1186/s12859-014-0423-x

**Published:** 2014-12-30

**Authors:** Jun Meng, Dong Liu, Chao Sun, Yushi Luan

**Affiliations:** School of Computer Science and Technology, Dalian University of Technology, Dalian, Liaoning 116023 China; School of Life Science and Biotechnology, Dalian University of Technology, Dalian, Liaoning 116023 China

**Keywords:** MiRNA, Pre-miRNA, Prediction, SVM, Feature selection

## Abstract

**Background:**

MicroRNAs (miRNAs) are a family of non-coding RNAs approximately 21 nucleotides in length that play pivotal roles at the post-transcriptional level in animals, plants and viruses. These molecules silence their target genes by degrading transcription or suppressing translation. Studies have shown that miRNAs are involved in biological responses to a variety of biotic and abiotic stresses. Identification of these molecules and their targets can aid the understanding of regulatory processes. Recently, prediction methods based on machine learning have been widely used for miRNA prediction. However, most of these methods were designed for mammalian miRNA prediction, and few are available for predicting miRNAs in the pre-miRNAs of specific plant species. Although the complete *Solanum lycopersicum* genome has been published, only 77 *Solanum lycopersicum* miRNAs have been identified, far less than the estimated number. Therefore, it is essential to develop a prediction method based on machine learning to identify new plant miRNAs.

**Results:**

A novel classification model based on a support vector machine (SVM) was trained to identify real and pseudo plant pre-miRNAs together with their miRNAs. An initial set of 152 novel features related to sequential structures was used to train the model. By applying feature selection, we obtained the best subset of 47 features for use with the Back Support Vector Machine-Recursive Feature Elimination (B-SVM-RFE) method for the classification of plant pre-miRNAs. Using this method, 63 features were obtained for plant miRNA classification. We then developed an integrated classification model, miPlantPreMat, which comprises MiPlantPre and MiPlantMat, to identify plant pre-miRNAs and their miRNAs. This model achieved approximately 90% accuracy using plant datasets from nine plant species, including *Arabidopsis thaliana*, *Glycine max*, *Oryza sativa*, *Physcomitrella patens*, *Medicago truncatula*, *Sorghum bicolor*, *Arabidopsis lyrata*, *Zea mays* and *Solanum lycopersicum*. Using miPlantPreMat, 522 *Solanum lycopersicum* miRNAs were identified in the *Solanum lycopersicum* genome sequence.

**Conclusions:**

We developed an integrated classification model, miPlantPreMat, based on structure-sequence features and SVM. MiPlantPreMat was used to identify both plant pre-miRNAs and the corresponding mature miRNAs. An improved feature selection method was proposed, resulting in high classification accuracy, sensitivity and specificity.

**Electronic supplementary material:**

The online version of this article (doi:10.1186/s12859-014-0423-x) contains supplementary material, which is available to authorized users.

## Background

MicroRNAs (miRNAs) are a family of non-coding RNAs approximately 21 nucleotides (nt) in length that play important roles at the post-transcriptional level in animals, plants and viruses [1]. These molecules are first cut from a stem-loop structure by RNaseDicer III. Environmental stress can induce or repress the expression of some miRNAs, thereby regulating the expression of downstream genes that respond to environmental stresses. The initial products of miRNA gene transcription are pre-miRNAs. Next, enzymes release pre-miRNAs with hairpin structures of 53–938 nt [2] by cutting and splicing. Finally, mature miRNAs are released from pre-miRNAs with hairpin structures by Dicer-like enzyme.

Mature miRNAs combine with RISC protein complexes to target specific mRNAs [3] and induce gene silencing by mRNA degradation or transcriptional inhibition. Plant miRNAs target multiple sites [4] to regulate various aspects of plant growth and development, including cell growth, cell differentiation, root, stem, leaf and other morphologies; these miRNAs also function in plant adaptation to different biotic and abiotic conditions [5,6].

The methods used to predict the role of miRNAs can be divided into two categories: experimental verification and bioinformatic prediction. Although experimental verification, which is based on direct cloning experiments, can identify many miRNAs with high expression levels, few miRNAs with low or specific expression can be identified. Moreover, this method is expensive and results in a high number of false positive results. Bioinformatic prediction can compensate for these deficiencies. Based on recent studies, bioinformatic methods for identifying miRNAs can be divided into three categories: alignment analysis, machine learning and high-throughput sequencing [7,8]. Studies have shown that miRNAs are conserved among species. Pre-miRNAs containing mature miRNAs can be folded to form hairpin structures that have low minimum free energy (MFE) values [9]. Alignment analysis is based on these properties. MiRscan [10], miRFinder [11] and miREval [12], which are based on alignment homology analysis, have been successfully applied. Due to a lack of miRNA structural information, most of these methods yield high false positive rates. Based on prior knowledge, appropriate data are selected, appropriate features are chosen, and a high-performance data-mining algorithm is used to construct a classification model. Triplet-SVM [13], bayesmiRNAfind [14] and MiPred [15] are successful models that are based on the machine learning method. However, few of these models can be used for plant pre-miRNA prediction because the hairpin structure of plant pre-miRNAs is much more complex than that of animal pre-miRNAs. Moreover, these models cannot be used to predict mature miRNAs in specific species [16]. High-throughput sequencing identifies not only pre-miRNAs but also mature miRNAs [17]. An integrated model to identify plant miRNA–target interactions has been proposed [18]. However, due to the existence of genome-wide sequencing errors, mistakes may occur when comparing with short sequences. Furthermore, some parameters are set based on experience and lack a strong theoretical basis. There is no consensus regarding miRNA prediction.

In this study, we focus on building a model that can be used in the classification of real/pseudo plant pre-miRNAs together with their mature miRNAs via the machine learning method. An initial set of 152 novel features related to sequential structure was used in the model. By applying feature selection, the subset of 47 features yielding optimal results was obtained using Back Support Vector Machine-Recursive Feature Elimination (B-SVM-RFE) in real/pseudo plant pre-miRNA classification. In the same way, the subset of 63 features yielding optimal plant miRNA classification was obtained. An integrated classification model, miPlantPreMat, was trained to identify real/pseudo plant pre-miRNAs and the corresponding miRNAs. MiPlantPreMat achieved high accuracy on plant datasets from nine plant species, including *Arabidopsis thaliana*, *Glycine max*, *Oryza sativa*, *Physcomitrella patens*, *Medicago truncatula*, *Sorghum bicolor*, *Arabidopsis lyrata*, *Zea mays* and *Solanum lycopersicum*. For example, 522 *Solanum lycopersicum* miRNAs were obtained from the *Solanum lycopersicum* genome sequence. The superior performance of the proposed classifier can be attributed to the extraction of plant pseudo pre-miRNAs, selection of the training dataset and careful feature selection. The website dedicated to miPlantPreMat includes the training and testing datasets, training models (MiPlantPre and MiPlantMat) and miPlantPreMat source codes used, all of which are freely available (https://github.com/kobe-liudong/miPlantPreMat). We provide a detailed description of the sources used for the datasets in the readme.txt in the ‘data’ folder.

## Methods

### Dataset preparation for the training and testing of the SVM model

An effective classifier of plant pre-miRNAs and miRNAs should distinguish real pre-miRNAs and miRNAs from pseudo pre-miRNAs and miRNAs. The positive dataset comprised known plant pre-miRNAs and miRNAs, whereas the negative dataset comprised pseudo *Solanum lycopersicum*, pseudo *Glycine max* and pseudo *Arabidopsis lyrata* hairpins.

**Figure 1:**
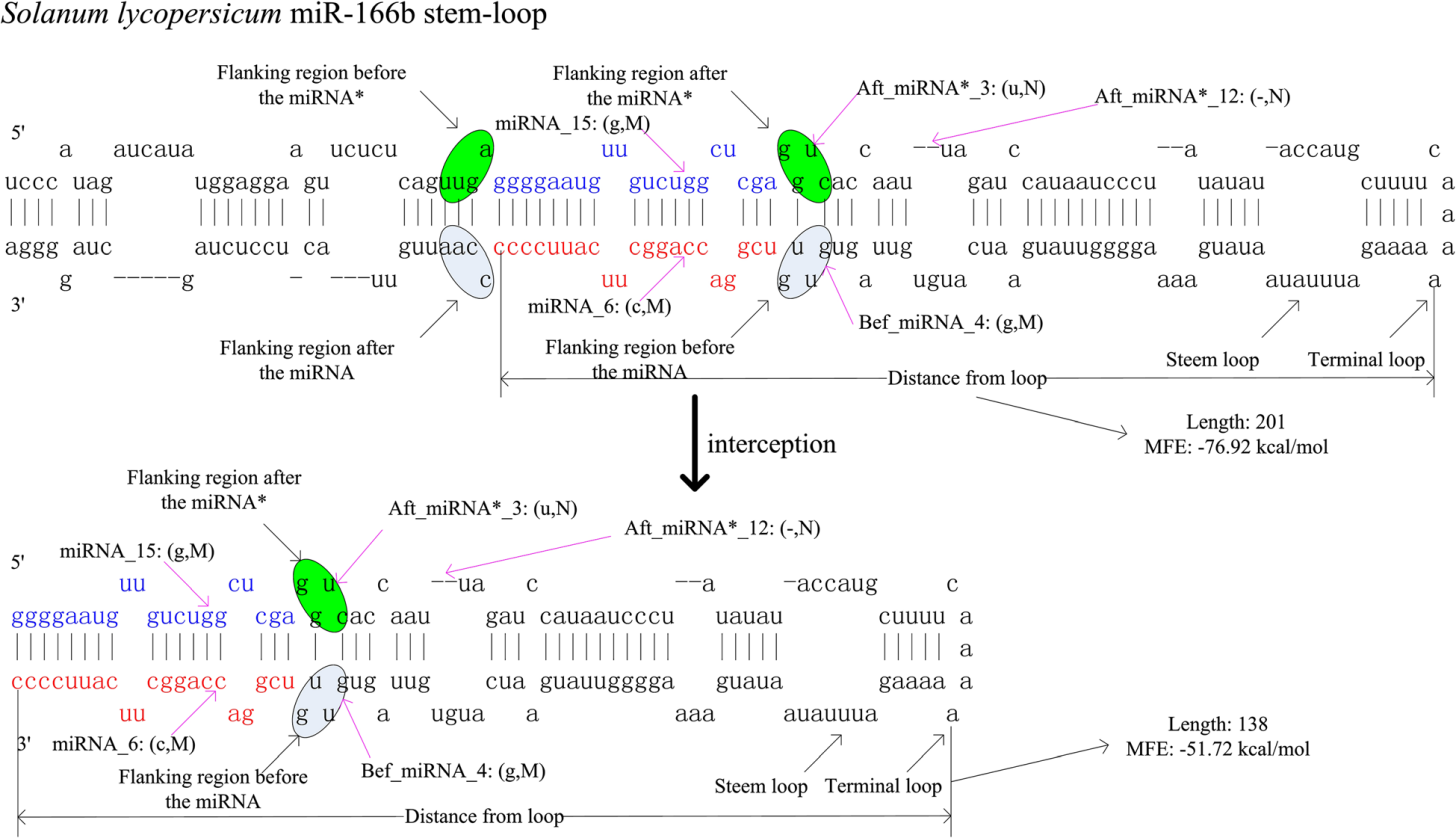
**Original pre**-**miRNA and intercepted pre**-**miRNA of** ***Solanum lycopersicum*** **miR**-**166b.** Mature miRNA is at 3’-end and miRNA* is at 5’-end of the selected sequence. Each base has two states, match or mismatch. Each precursor contains one loop at least. The original pre-miRNA has 201 bases with the MFE −76.92 kcal/mol and the intercepted pre-miRNA has 138 bases with the MFE −51.72 kcal/mol.

All 6,378 plant miRNAs and 5,166 plant pre-miRNAs that were experimentally verified in miRBase release 19.0 were screened for inclusion in the positive data set. Redundant sequences were excluded, and the remaining non-redundant sequences were folded into hairpin secondary structures. Of these pre-miRNAs, 3,126 non-redundant pre-miRNAs having single stem-loops were treated as real samples for miPlantPre. Plant pre-miRNAs range from 53 nt to 938 nt in length and have more complex secondary structures than mammalian pre-miRNAs. It is difficult to locate the position of miRNAs and miRNAs* for plant pre-miRNAs. In this study, pre-miRNAs were intercepted such that mature miRNAs in pre-miRNAs are at the 3’- or 5’-end of the selected sequence. We treated these pre-miRNAs as real samples within miPlantMat. Furthermore, pre-miRNAs intercepted at other positions were treated as pseudo samples within miPlantMat. After interception, the pre-miRNAs exhibited a narrower length range, and structures in each region of the pre-miRNAs were more unified. The 152 features applied in miPlantPre were also used because the stem-loop structure was maintained. In this process, the proposed features termed MFEI7, MFEI8, MFEI9, Mis_num_begin, Mis_num_end and "G(((_begin_S", "A.(._end_S" were useful because they helped to identify real pre-miRNAs that were intercepted at different positions. For consistency, all pre-miRNA secondary structures were recalculated using RNAfold in the Vienna package [19]. Figure 1 illustrates the interception procedure using the stem-loop of *Solanum lycopersicum* miR-166b as an example. The length was shortened from 201 nt to 138 nt by removing the bases before the miRNA* and after the miRNA.

Almost all reported miRNAs are located in untranslated regions or intergenic regions, although some can be found in protein-coding sequences (CDSs). Some sequence segments have stem-loop structures that are similar to those of real pre-miRNAs but have not been reported as pre-miRNAs. Because the model was trained to distinguish analogous real/pseudo pre-miRNAs, the sequences in the negative dataset should regard structures with stem-loop structures as genuine pre-miRNAs; otherwise, the classification will not yield significant results. CDSs of *Solanum lycopersicum*, *Glycine max* and *Arabidopsis lyrata* RefSeq genes with no known alternative splice events were collected. Most known plant pre-miRNAs are 120 nt in length. Thus, a sliding window of widths ranging randomly from 60 to 150 nt was used to scan the CDSs to produce sequence segments. The sequence segments should fold into single stem-loop structures and satisfy five criteria based on the number of base pairs in hairpins, %G + C, MFEI, the complementary base pairing of mature miRNAs and the stability of the precursor in relation to the MFE rate. The criteria were determined by observing real intercepted plant pre-miRNAs. The criteria for selecting pseudo miRNAs were as follows: a minimum of 19 base pairings in the hairpin structure, %G + C > 0.242 and <0.825, MFEI >0.522 and <1.39, no multiple loops, at most 3 continuous unpaired bases and at most 7 unpaired bases in the mature miRNAs. All precursor secondary structures were recalculated with p-values of 0.01 and the ‘-p’ option at 37°C. The frequency distribution of MFE and the empirical distribution were modeled using a modified sigmoid function

$$ x=MFE/ length $$

$$ f(x)=\frac{a}{b+{e}^{x*c}} $$

Where *length* represented the length of the precursor, *a* = 1.339e-12, *b* = 2.7783e-13, and *c* = 45.843 were the fitting parameters. The stability was calculated using *f*(*x*). The selection criterion was *f*(*x*) > −4.42. Finally, 8,494 pseudo pre-miRNAs were collected as the negative dataset.

While training the model miPlantMat, we collected pre-miRNAs that were not intercepted by mature miRNAs or miRNAs* from the primary pre-miRNAs. We treated these pre-miRNAs as pseudo samples for miPlantMat. The sequences either contained real samples for miPlantMat or were contained in real samples for miPlantMat. If a base was paired with another base on the opposite strand of the stem in the pseudo pre-miRNAs, the paired base was collected in the pre-miRNAs to maintain the stem-loop structure. Consequently, the pseudo samples must be similar to the real samples for the classification to be significant.

### Features of plant miRNAs and pre-miRNAs

Recent studies have demonstrated that the primary sequence and secondary structure of plant pre-miRNAs exhibit many features that can be used to classify real/pseudo plant pre-miRNAs. Because the sequences of almost all mature miRNAs are located in the stems of the corresponding pre-miRNAs, the sequences either begin from miRNAs and end in miRNAs or form a stem-loop structure. Based on these features, mature miRNAs can be located in pre-miRNAs. The stem-loop of *Solanum lycopersicum* miR-166b was used as an example. The stem-loop without interception was treated as a real sample in the MiPlantPre model of miPlantPreMat. The stem-loop with interception is treated as a real sample in the miPlantMat model of miPlantPreMat.

Structural characteristics are also very important for identifying real/pseudo pre-miRNAs. 32 structured triplet composition features are defined in triplet-SVM (including the frequencies of “G(((“ and ”C((.“, which are extracted from the pre-miRNAs. A left bracket ”(“ indicates that a paired nucleotide is located near the 5’-end and can be paired with another nucleotide at the 3’-end, and the corresponding nucleotide at the 3’-end is indicated using a right bracket ”)“. As in previous studies, ”(“ and ”)“ were treated equally. A dot ”.“ indicates that a nucleotide does not pair with a nucleotide on opposing end. These 32 features were extracted from stems and are denoted as ”G(((_S“ and ”C((._S“, etc.

29 global and intrinsic folding features were extracted from secondary structures of real/pseudo pre-miRNAs defined in miPred. These features include the following: (i) %G + C content and 16 dinucleotide frequencies defined as %XY, where X, Y in {A, C, G, U}; (ii) adjusted base pairing propensity denoted as dP [20]; (iii) the MFE of folding denoted as dG [21]; (iv) the adjusted base pair distance denoted as dD [22]; (v) the adjusted Shannon entropy denoted as dQ [23]; (vi) the MFE index denoted as MFEI1 and MFEI2 [24], a topological descriptor of the degree of compactness denoted as dF; and (vii) 5 normalized variants of dP, dG, dQ, dD and dF denoted as zP, zG, zQ, zD and zF, respectively [25].

19 features defined in microPred [26] include the following: (i) seven base pair-related features that are denoted as |A − U|/L, |G − C|/L, |G − U|/L, the average number of base pairs per stem (Avg_BP_Stem), %(A − U)/n_stems, %(G − C)/n_stems and %(G − U)/n_stems; (ii) the MFE index denoted as MFEI3 and MFEI4; (iii) four RNA fold-related features, such as the normalized ensemble free energy (NEFE); the frequency of the MFE structure denoted as Freq; structural thermodynamic features such as the structural entropy dS and dS/L; the structural enthalpy dH and dH/L; and the melting energy of the structure, denoted as Tm and Tm/L, where L represents the length of the pre-miRNA sequences and n_stems represents the number of stems in the secondary structure.

3 features defined in PlantMiRNAPred [27] include: (i) the MFE index denoted as MFEI5 and MFEI6; (ii) the average number of mismatches per 21-nt window, which is calculated as Avg_mis_num = tot_mismatches/n_21nts, where tot_ mismatches is the total number of mismatches in the 21-nt sliding window and n_21nts is the number of sliding windows in a stem.

69 novel features proposed in our study include the following: (i) MFE Index 7: MFEI7 = MFE/%G + C_ Begin_n_ 21nts, where %G + C_ Begin_ n_21nts is the GC content in the first 21 bases of the stems; MFE Index 8: MFEI8 = MFE/%G + C_End_n_21nts, where %G + C_End_n_21nts is the GC content in the last 21 bases of the stems; MFE Index 9: MFEI9 = MFE/avg_ mis_num_n_21nts, where avg_mis_num_n_21nts is the average number of mismatches per 21-nt window; (ii) Mis_ num_begin: the nucleotide is not paired with a nucleotide on the opposing terminus in the first 21 bases of the stems; (iii) Mis_num_end: the nucleotide is not paired with a nucleotide on the opposing terminus in the last 21 bases of the stems. Because the miRNAs and miRNAs* are stable, it is necessary to determine the position of the mature miRNAs in the corresponding pre-miRNAs; and (iv) to obtain improved classification, features that reflect both the sequence and secondary structure of the real/pseudo pre-miRNAs and that aid in determining the position of the mature miRNA in the pre-miRNA were needed. In addition to the features extracted above, 64 new features including the frequencies of ”G(((_begin“ and ”A.(._end“ were extracted from the beginning and end of pre-miRNAs. Because almost all mature miRNAs were located in stems, these 64 features were extracted from stems and were denoted as ”G(((_begin_S“ and ”A.(._end_S“, etc.

152 features belonging to six groups were selected, as shown in Table 1. MFEI1, MFEI2, MFEI3, MFEI4, MFEI5, MFEI6, MFEI7, MFEI8, and MFEI9 were considered MFE-related features. 20 features that reflect the proportion of adjacent bases and the G and C content of bases were used as sequence-related features. 6 thermodynamic features were used as mfold-related features. Seven types of base pairing were used as base pair-related features. 96 features were triple-related. 14 features calculated by RNAfold were used as RNAfold-related features. Secondary structures and thermodynamic parameters were obtained using the ViennaRNA package. All RNAfold-related features were extracted using the RNAfold program using the ‘-p’ option at 37°C. For consistency, every parameter was scaled in the range from −1 to 1.

**Table 1:** **Selected pre**-**miRNA features**

**Classification**	**Number**	**Features**
MFE-related	9	MFEI1^2^, MFEI2^2^, MFEI3^3^, MFEI4^3^, MFEI5^4^, MFEI6^4^, MFEI7^5^, MFEI8^5^, MFEI9^5^
Sequence-related	20	%AA,%AC, etc.^2^ (16),%G + C^2^, Avg_mis_num^4^ Mis_num_begin^5^, Mis_num_end^5^
Mfold-related	6	dS^3^, dS/L^3^, dH^3^, dH/L^3^, Tm^3^, Tm/L^3^
Base-pair -related	7	|A-U|/L^3^,|C-G|/L^3^, |G-U|/L^3^, Avg_BP_Stem^3^, %(A − U)/n_stems^3^, %(G − C)/n_stems^3^
Triple-related	96	A(((_S, A((._S, etc.^1^ (32), A(((_begin_S, A((._begin _S, etc.^5^ (32), A(((_end _S, A((._end _S, etc.^5^ (32)
RNAfold-related	14	dP^2^, dG^2^, dD^2^, dQ^2^, dF^2^, zP^2^, zG^2^, zD^2^, zQ^2^, zF^2^,NEFE^3^, Freq^3^, Diversity^3^, Diff^3^

^1^Features extracted in triplet-SVM.

^2^Features extracted in miPred.

^3^Features extracted in microPred.

^4^Features extracted in plantMiRNAPred.

^5^Features extracted in miPlantPreMat.

### SVM and miPlantPreMat classifier

We chose SVM as our classification paradigm in this research based on its excellent generalization ability. For a given dataset *X*_*n*_, *x*_*i*_ ∈*X*_*n*_ (*i* = 1, 2,…, *N*), each element in the dataset has a corresponding label *γ*_*i*_ (−1 or +1, representing the two classes to be classified; +1 represents real samples whereas −1 represents pseudo samples). A decision function is given by the SVM classifier

$$ f(x)=\operatorname{sgn}\left({\displaystyle \sum_{i=1}^N{\gamma}_i{\alpha}_iK\left(x,{x}_i\right)+b}\right) $$

Where *γ*_*i*_ is the class label of the *i*-th element, *α*_*i*_ is the coefficient to be learned, *K* is the kernel function, and *b* is the offset. *α*_*i*_ is obtained by maximizing

$$ {\displaystyle \sum_{i=1}^N{\alpha}_i-\frac{1}{2}{\displaystyle \sum_{i,j=1}^N{\alpha}_i{\alpha}_j{\gamma}_i{\gamma}_jK\left({x}_i,{x}_j\right)}} $$

If the value of *f*(*x*) is greater than zero, the label assigned to data *x* is +1; otherwise, the assigned label is −1.

The LIBSVM package (version 3.1) [28] was used in our study. To obtain the best performance, the penalty parameter *C* and the RBF kernel parameter *γ* were calculated using grid search strategy.

**Figure 2:**
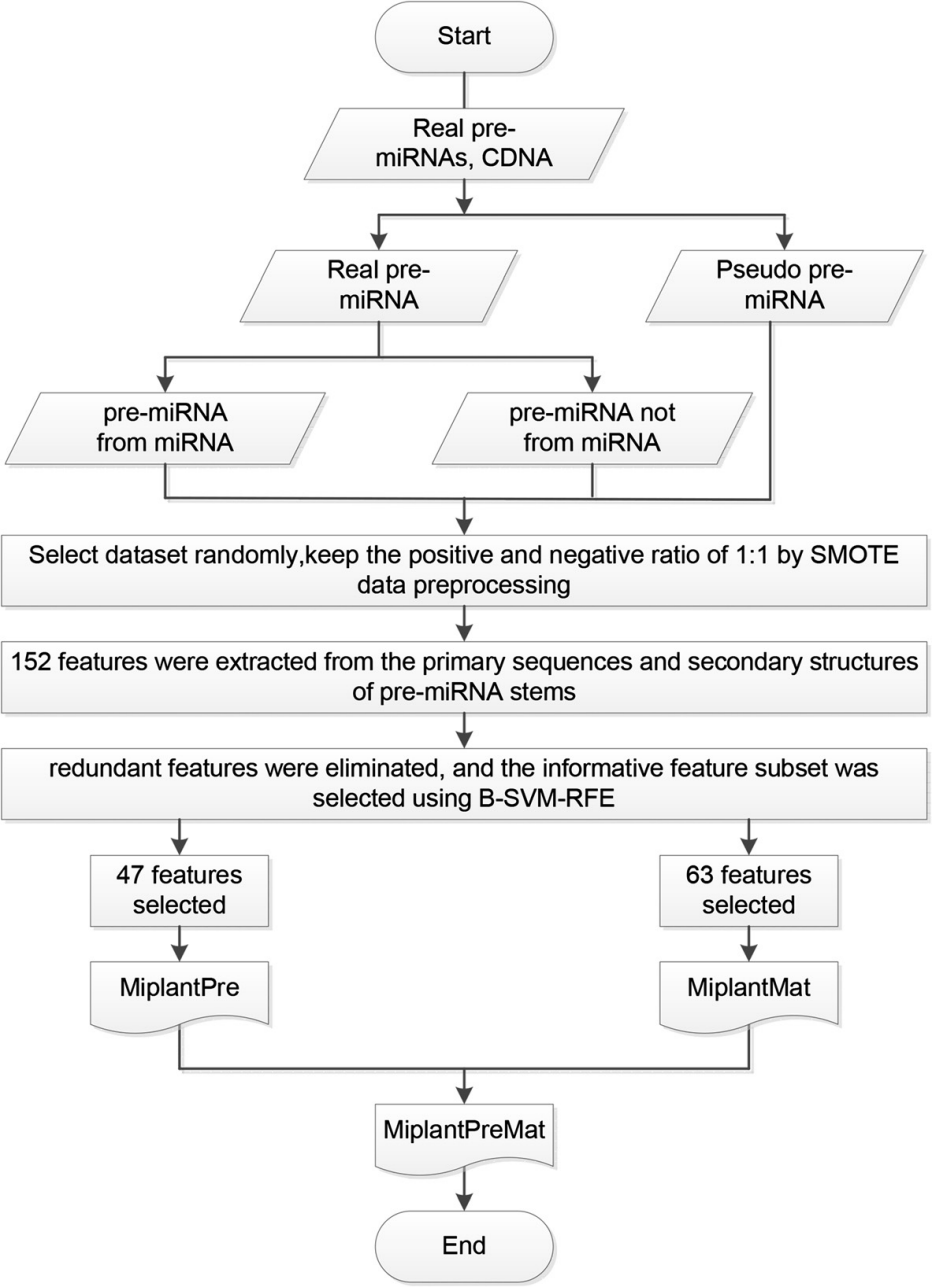
**Flow chart of the classification model miPlantPreMat for use with plant miRNAs.** Construction of SVM classifier MiPlantPreMat based on feature selection and sample selection was shown.

MiPlantPreMat was proposed based on SVM, as illustrated in Figure 2. A total of 3,126 non-redundant plant pre-miRNAs with single stem-loops were collected from miRBase release19.0 and used as the positive dataset. A total of 8,494 non-redundant sequence segments with stem-loop structures similar to real pre-miRNAs that were not previously reported as pre-miRNAs were collected and used as the negative dataset. (i) A total of 2,000 positive and 2,000 negative samples were randomly collected for use in training the miPlantPre model of MiPlantPreMat; (ii) 152 features were extracted from the primary sequences and secondary structures of pre-miRNA stems; (iii) redundant features were eliminated, and the informative feature subset was selected using B-SVM-RFE; (iv) miPlantPre was trained with the selected 47 features; (v) 3,835 sequence segments from the 3,126 pre-miRNAs mentioned above were collected and used as the positive dataset. The sequence segments extended from the beginning of the mature miRNAs to the end of the miRNAs*, from both the 5’ and 3’ arms. A total of 39,428 sequence segments from the same pre-miRNAs, which were not previously included in the positive dataset, were longer than 55 nt, and comparable stem-loop structures were collected for use as the negative dataset. Randomly, 1,000 positive and 5,000 negative samples were collected and preprocessed using SMOTE to train the miPlantMat model of MiPlantPreMat, keep the positive and negative ratio of 1:1; (vi) miPlantMat was trained using the selected 63 features using the same method; and (vii) an integrated MiPlantPreMat model was constructed by combining MiPlantPre and MiPlantMat. The detailed feature extraction and selection of the SVM model are shown in Additional file 1.

### Feature subset selection

Feature subset selection is used to choose a group of informative features that retain the most information from the original data, screen out redundant features and distinguish each sample in the dataset. A total of 152 features were selected without considering redundancy and correlation with class. SVM-RFE [29,30] was used for subset selection in our study.

SVM-RFE is a simple and efficient feature selection algorithm that ranks features according to the SVM classification results. The evaluation function is biased toward subsets that contain features that are highly correlated with class. Irrelevant features should be ignored because they will be poorly correlated with class. Feature subset selection can be summarized as follows: (i) input training examples *X*_0_ = [*x*_1_, *x*_2_, …, *x*_*n*_]^T^ together with their class labels *y* = [*y*_1_, *y*_2_, …, *y*_*n*_]^T^; (ii) initialize the subset of surviving features *s* = [1, 2, …, 152] and the features ranked list *r* = [], repeat until *s* = []; (iii) restrict the training examples to those exhibiting good feature indices *X* = *X*_0_(:, *s*) and train the classifier *α* = *SVM*-*train*(*X*, *y*); (iv) compute the weight vector of dimension length(*s*), $w={\displaystyle \sum_k{\alpha}_k{y}_k{x}_k}$; (v) compute the ranking criteria *c*_*i*_ = (*w*_*i*_)^2^ for all *i*; (vi) find the feature with the smallest ranking criterion *f* = *argmin*(*c*) and update the feature ranked list *r* = [*s*(*f*), *r*]; (vii) eliminate the feature with the smallest ranking criterion *s* = *s*(1: *f*-1, *f* + 1: length(*s*)); and (viii) find the classifier *α* and the subset of trained classifiers *α*.

A total of 152 features without redundancy were extracted under the initial conditions. These features represent a sample but do not fully consider the relationship between the attributes during extraction and classification. SVM-RFE can dynamically calculate attribute weights, sort each attribute, and fulfill feature selection. However, once the attributes are sorted to the bottom, they can no longer participate in subsequent attribute weight calculations. Because the training number is different each time, the properties calculated under different SVM classification space attribute weights also differ. Therefore, sorting of the calculated weights of less important properties may be overshadowed by properties with a higher weight. Here, we propose the use of a B-SVM-RFE method that is based on the attribute of Information Gain [31] (IG).

Information entropy is an important concept underlying information gain. For a classification system, the possible values of a category are *C*_1_, *C*_2_,…, *C*_*n*_, where *P*(*C*_1_), *P*(*C*_2_), …, *P*(*C*_*n*_) represent the probabilities of each category and *n* represents the total number of categories. The information entropy of the classification system is expressed as:

$$ H(C)=-{\displaystyle \sum_{i=1}^nP\left({C}_i\right)* \log {}_2P\left({C}_i\right)} $$

Information gain is reliant on characteristic *t*. When calculating the differences in information entropy between when characteristic *t* exists and when it does not, the increased amount of information obtained is the information gain.

Characteristics of *t* included in the system of information entropy can be obtained. When *t* does not belong to the system, feature *t* is treated as a constant. Then, the problem can be seen as computing the conditional entropy with constant *t*

$$ H\left(C\Big|T\right)=P(t)H\left(C\Big|t\right)+P\left(\overset{-}{t}\right)H\left(C\Big|\overset{-}{t}\right) $$

Where *T* is the characteristic, *t* indicates the presence of characteristic *T*, and $\overset{-}{t}$ indicates the absence of characteristic *T*. Then, the information gain of characteristic *T* can be calculated as follows:

$$ IG(T)=H(C)-H\left(C\Big|T\right)={\displaystyle \sum_{C,T}p(CT) \log {}_2\frac{p(CT)}{p(C)p(T)}} $$

A total of 2,000 real samples and 2,000 pseudo samples were chosen from the data pool using progressive sampling. The information gain and SVM-RFE ranking of the 4,000 samples regarding the 152 features are listed in Table 2.

**Table 2:** **Information gain of each attribute and SVM**-**RFE ranking**

**Feature**	**IG**	**SVM**-**RFE rank**	**Feature**	**IG**	**SVM**-**RFE rank**
*dP*	0.78628	1	*U*…_*S*	0.09652	58
*MFEI5*	0.77982	2	*C*…_*S*_*end*	0.0933	103
*zP*	0.75613	3	*G*(((_*S*	0.07866	30
*MFEI7*	0.68656	54	*A*…_*S*_*end*	0.07662	74
*MFEI8*	0.66704	48	*C*(((_*S*	0.072	13
…	…	…	…	…	…
%*GG*	0.12375	38	*G*(((_*S*	0.07866	30
*MFEI6*	0.1227	25	*A*…_*S*_*end*	0.07662	74
*dH*/*L*	0.11855	77	*C*(((_*S*	0.072	13
%*CU*	0.11651	8	%(*G*-*C*)/*n*_*stems*	0.07079	44
*MFEI4*	0.11603	15	*G*…_*S*_*begin*	0.06746	93
*G*…_*S*_*end*	0.11563	34	%*GC*	0.06041	28
*C*.(._*S*	0.11034	139	*U*.(._*S*	0.05969	101
*dF*	0.10372	127	*A*…_*S*_*begin*	0.05779	53

**Figure 3:**
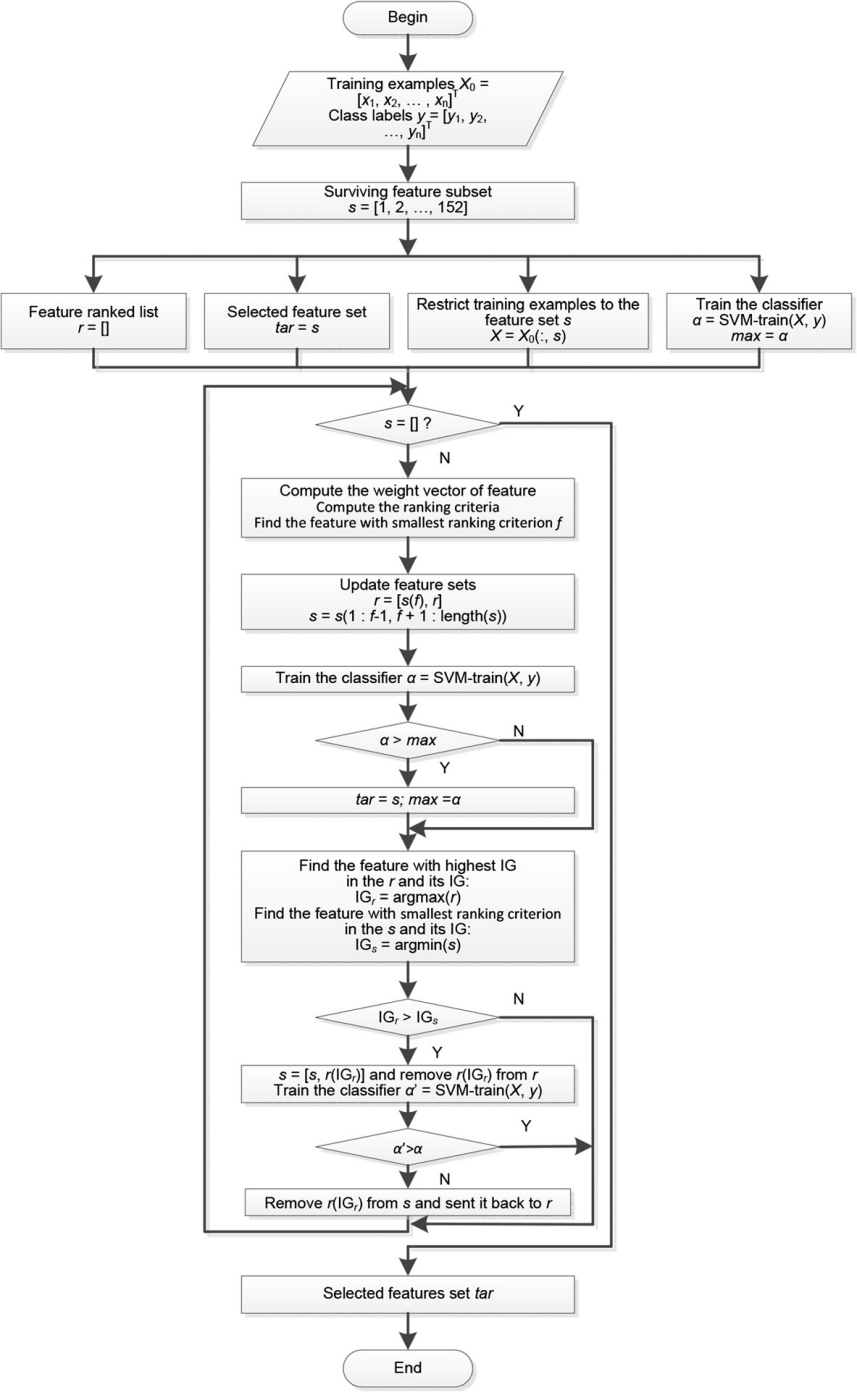
**Flow chart of B**-**SVM**-**RFE feature selection.** Feature subset was selected using B-SVM-RFE. This method was combined by SVM-RFE and information gain. The final feature subset for miPlantPreMat was obtained.

First, the information gain of each attribute and the SVM-RFE ranking were calculated. Then, the existing set *s* = [1, 2, …, 152] and ranking set *r* = [] of the properties were updated. The SVM model was trained, and the property ranking was sent to ranking set *r*. If properties existed that yielded higher information gain than the property with the lowest weight in *s*, then the property with the highest information gain in *r* would be sent to the existing set *s*. Then, the SVM model was trained again. If the cross validation error at this time was better than that obtained during the previous run, then the property with the highest information gain in *r* would be sent back to *s*. The existing set and the ranking set would be updated and used to train the SVM model again. If the cross validation error at this time was not better than that obtained during the previous run, then the property would be sent back to *r*. The SVM model was trained until no property was present in existing set *s*. Finally, the property set with the best cross validation error was selected for use. The process is illustrated in Figure 3.

**Figure 4:**
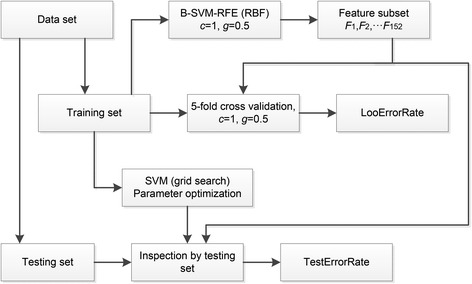
**Determination of the best feature subset.** Two indicators named LooErrorRate and TestErrorRate were used for the best subset evaluation. The LooErrorRate was calculated with 5-fold cross validation model. The TestErrorRate was calculated by independent training set and testing set with optimized parameters. The parameters of penalty coefficient *c* and the kernel function parameter *g* were obtained by grid search method.

During feature selection, the 5-fold cross validation recognition rate (LooErrorRate) and independent test error recognition rate (TestErrorRate) were used to determine the best feature set. When B-SVM-RFE was used to train the model with 5-fold cross validation, the parameter of the penalty coefficient *C* and the kernel function parameter *g* were set to the default values. When tested using an independent test set, the grid search method was used to determine the best parameters. The process used to determine the best feature set is shown in Figure 4.

In this paper, 2,000 real samples and 2,000 pseudo samples were used to train the SVM model; 1,000 real samples and 1,000 pseudo samples were used in the test set, and the principle of the fence was used to verify that no sample appeared repeatedly both in the training and testing sets. The feature set *F*_1_, *F*_2_, …, *F*_152_ represents the number of corresponding properties of the sample space selected using B-SVM-RFE. The best classification rate was obtained using this feature subset. The classification rate was tested using 5-fold cross validation, and the LooErrorRate and TestErrorRate for SVM-RFE and B-SVM-RFE obtained in this experiment are provided in Table 3. The lowest 5-fold cross validation recognition rate (LooErrorRate) and the independent test error recognition rate (TestErrorRate) were 2.42% and 7.04%, respectively. In this paper, this subset of 47 features was selected to train miPlantPreMat.

**Table 3:** **LooErrorRate and TestErrorRate of SVM**-**RFE and B**-**SVM**-**RFE**

**Feature number**	**SVM**-**RFE**		**B**-**SVM**-**RFE**	
	**LooErrorRate**	**TestErrorRate**	**LooErrorRate**	**TestErrorRate**
1	21.13	26.53	21.13	26.53
2	11.40	21.01	11.40	21.01
3	9.91	20.94	9.91	20.94
…	…	…	…	…
46	3.04	7.15	2.72	7.15
47	2.84	7.34	2.42	7.04
48	2.72	7.14	2.72	7.14
…	…	…	…	…
150	3.00	8.17	3.00	8.17
151	3.19	8.29	3.19	8.29
152	3.30	7.30	3.30	7.30

Normal plant pre-miRNAs are 60–150 nt in length. Only one miRNA is located in the pre-miRNA; however, more than one pseudo miRNA can be obtained from the pre-miRNA. If the same positive and negative data rates were to be applied to miPlantMat and miPlantPre, the obtained information might reflect pseudo samples from miPlanMat that would be of little use to the classification model and possibly increase the false positive rate. In this paper, the positive and negative data rates were set at 1:5, indicating that if one positive data sample were selected, then five negative data samples would also be selected. This data rate is closer to that of the original data samples and reflects the distribution features of the data samples. However, this result illustrates the class imbalance problem. The data classification is biased toward the negative class, potentially resulting in a high false negative rate. To solve these problems, the SMOTE [32] method was used for data processing.

Two potential methods can be used to solve the classification problem for unbalanced data. The first method is to balance the dataset, and the second is to improve the performance of the machine learning algorithm on specific issues. In 2002, Chawla proposed a method that improved the fitting sample problem caused by traditional classification. The main idea behind SMOTE was to increase rare class samples by joining the closer rare class samples to the ”simulation“ samples and then increasing the number of rare class samples to approach a dense sample number. The specific experimental steps are as follows:

- i. determine the sampling ratio *N* and the number of rare class samples *x*; identify *k* nearest neighbors for each rare class sample; identify *N* points from the original *x* class samples and their x*k similar samples;
- ii. identify each *k* nearest neighbors from the selected *N* rare class samples and the original rare class samples, and then identify *k* neighbor samples of the (*x* + *N*)*k class samples from the original sample; new rare class samples randomly generate *N* points as the rare generated class sample;
- iii. new rare class samples are added to the original training set, thus forming a new training data set,

$$ {x}_{new}=x+ rand*\left(y\left[i\right]-x\right) $$

Where *i* = 1, 2, …, *N*; *rand* is a random number between 0 and 1; *x*_*new*_ represents the new sample; *x* represents the original sample; and *y*[*i*] represents the *i*-th neighbor of *x*.

### Evaluation method

The classification model was trained using the informative feature subset and the training samples using 5-fold cross-validation and default values for *g* and *C*. Datasets were optimized for *g* and *C* using the grid selection approach recommended by LIBSVM. The accuracy of the prediction result was evaluated based on the number of true positives (*TP*), false positives (*FP*), true negatives (*TN*) and false negatives (*FN*). The sensitivity (*SE*), specificity (*SP*), geometric mean (*Gm*) and total prediction accuracy (*Acc*) used to assess the prediction system were calculated according to the following definitions:

$$ SE=\frac{TP}{TP+FN} $$

$$ SP=\frac{TN}{FP+TN} $$

$$ Acc=\frac{TN+TP}{TP+FP+FN+TN} $$

$$ Gm=\sqrt{SE\times SP} $$

Where *SE* is the proportion of positive samples (real pre-miRNAs) that are correctly classified as pre-miRNAs, and *SP* is the proportion of negative samples (pseudo pre-miRNAs) that are correctly classified as pre-miRNAs.

## Results

### The results of feature subset selection

To obtain the highest classification performance, three subset selection methods were used in this paper: Principal Components Analysis (PCA), Correlation-based Feature Subset Selection (CFS) [33] and B-SVM-RFE. Additionally, three machine-learning methods were used in this paper: naiveBayes (NBC) [34], RandomForest (RF) [35] and SVM. Finally, the subset collected using B-SVM-RFE and trained using SVM was chosen because it performed better than the other selection methods. Subsets containing 47 features used for miPlantPre and 63 features used for miPlantMat were acquired. The selected features were ranked as shown in Additional files 2 and 3. Among the selected features, dS, dH and Tm are related to mfold. A number of studies have verified that the stem-loop structures of plant pre-miRNAs are thermodynamically stable [36]. A(((_S, G…_S and C…_S are triple-related. Studies have shown that local adjacent structures can be applied to distinguish real pre-miRNAs from pseudo miRNAs [37]. The features %AA, %UC and %G + C are related to sequence. Because pre-miRNAs are composed of nucleotide sequences that have unique characteristics, the sequence composition of pre-miRNAs is useful for classification [12].

Table 4 shows that the SVM using subset selection method B-SVM-RFE has the best performance.

**Table 4:** **Classification results based on different feature subsets using three methods**

**Model**	**ML method**	**Feature subset selection method**	**Feature number**	**Classification results (%)**			
				***SE***	***SP***	***Acc***	***Gm***
miPlantPre	NBC	PCA	76	92.2	92.6	92.4	92.4
		CFS	20	93.9	97.8	95.8	95.8
		B-SVM-RFE	47	93.8	98.6	96.2	96.2
		All features	152	92.9	98.0	95.4	95.4
	RF	PCA	76	93.5	95.3	94.4	94.4
		CFS	20	95.0	97.6	96.3	96.3
		B-SVM-RFE	47	95.3	97.7	96.5	96.5
		All features	152	95.3	97.7	96.5	96.5
	SVM	PCA	76	94.9	99.2	97.0	97.0
		CFS	20	94.3	99.1	96.7	96.7
		B-SVM-RFE	47	95.5	99.1	97.2	97.2
		All features	152	93.9	98.5	96.2	96.2
miPlantMat	NBC	PCA	71	88.6	82.3	85.5	85.4
		CFS	40	93.2	74.8	83.6	83.5
		B-SVM-RFE	63	89.8	88.4	89.1	89.1
		All features	152	91.7	79.3	85.5	85.3
	RF	PCA	71	93.2	73.2	83.2	82.6
		CFS	40	89.2	89.1	89.2	89.2
		B-SVM-RFE	63	89.7	88.6	89.2	89.2
		All features	152	86.6	84.4	85.5	85.5
	SVM	PCA	71	88.6	84.3	86.4	86.4
		CFS	40	90.6	87.5	89.1	89.1
		B-SVM-RFE	63	92.9	88.7	90.8	90.8
		All features	152	87.1	81.6	84.4	84.4

### Parameter subset selection compared with other methods

To test the efficiency of our model, we compared miPlantPre with five existing models (Triplet-SVM, MiPred, miPred, miRabela and microPred). Table 5 shows that miPlantPre exhibited better performance than the existing models in terms of sensitivity (*SE*), specificity (*SP*), geometric mean (*Gm*) and total prediction accuracy (*Acc*) while using fewer features.

**Table 5:** **Comparison of miPlantPre against other methods**

**Methods**	**Training dataset**		**Testing dataset**		**Features selected**	**Classification results (%)**			
	**pos**	**neg**	**pos**	**neg**		***SE***	***SP***	***Acc***	***Gm***
Triplet-SVM	163	168	30	1000	32	93.30	88.10	90.66	90.66
MiPred	163	168	263	265	34	89.35	93.21	91.26	91.26
miPred	200	400	123	146	34	84.55	97.97	91.01	91.01
miRabela	*Not given clearly in the article*					71.00	97.00	82.99	82.99
microPred	*SMOTE* + *outer*-*5*-*fold*-*cv*				21	90.02	97.28	93.58	93.58
plantMiRNAPred	*outer*-*5*-*fold*-*cv*				68	91.93	97.84	94.84	94.84
miPlantPre	*outer*-*5*-*fold*-*cv*				47	95.50	98.82	97.16	97.16

### Tests on different plant species

Pre-miRNAs of *Arabidopsis thaliana* (ath), *Glycine max* (gma), *Oryza sativa* (osa), *Physcomitrella patens* (ppt), *Medicago truncatula* (mtr), *Sorghum bicolor* (sbi), *Arabidopsis lyrata* (aly), *Zea mays* (zma) and *Solanum lycopersicum* (sly) were used to compare the efficiency of miPlantPre with three widely used methods. To show that the false positive rate was sufficiently low, a negative dataset was used to test the efficiency of miPlantPre (Table 6).

**Table 6:** **The classification accuracy of four methods for the pre**-**miRNA of several plants species and for the negative dataset**

**Plant species & negative dataset**	**Methods**			
	**Triplet**-**SVM**	**MiPred**	**plantMiRNAPred**	**miPlantPreMat**
aly	94.29	96.19	96.19	99.05
ath	91.75	90.72	92.78	96.91
gma	91.18	92.65	93.93	95.89
mtr	85.90	88.46	89.74	90.60
osa	92.31	95.10	95.10	95.10
ppt	88.44	91.16	97.96	98.64
sbi	93.38	97.79	96.99	98.53
sly	97.14	100.00	100.00	100.00
zma	89.74	97.44	97.44	98.29
neg	94.80	97.80	98.20	98.60

miPlantPre performed better than Triplet-SVM and microPred for most species. The sub-sequences in pre-miRNAs that begin from the miRNAs and end at the miRNAs or that form a stem-loop structure were selected. These nine species were also used to test the efficiency of miPlantMat regarding miRNA classification. The results are shown in Table 7. These pre-miRNAs and miRNAs were published in miRBase release 20.0.

**Table 7:** **The classification results obtained using miPlantMat for various pre**-**miRNA datasets**

**Plant species**	**Classification results (%)**	
	**Accuracy**	**FPR**
aly	89.46	9.46
ath	87.84	10.53
gma	89.50	13.36
mtr	87.67	12.22
osa	88.96	10.31
ppt	90.98	10.46
sbi	89.02	9.53
sly	89.87	8.36
zma	91.42	10.93

The accuracies found using these species were all greater than 87%, demonstrating the utility of miPlantMat for classification in plants. Moreover, the false positive rates (FPRs) obtained were all lower than 13.36%.

### Searching miRNAs in *Solanum lycopersicum*

Studies have shown that miRNAs are relatively conserved during the evolutionary process. Therefore, some miRNAs exhibit conservative evolutionary relationships among species [38]. There are two basic principles behind our method. One is that homologous fragments can be identified according to sequence or structural similarity. The second is that new miRNAs can be discovered using known miRNAs [39]. To date, 77 *Solanum lycopersicum* mature miRNAs have been reported in miRBase (Release 21, 2014.6.26). Through studying these 77 miRNAs, which are distributed among 31 miRNA families.

MiRNAs of the same family may be found in a large number of species. In this study, known plant miRNAs were used to identify potential miRNAs in *Solanum lycopersicum*. First, genome-scale fragments might contain similarities to known miRNAs. If less than 3 mismatches were found for two related sequences, we considered the sequences similar. The KMP [40] algorithm was used to compare known miRNAs on a genome-wide scale. A series of potential miRNAs was obtained for comparison. Structural information regarding putative miRNAs was obtained using RNAfold. Potential pre-miRNAs were obtained by identifying stem-loop-containing fragments. Several potential miRNAs with hairpins were obtained by limiting the minimum number of base pairings in the hairpin structure to 19, %G + C > 0.242 and <0.825, MFEI >0.522 and <1.39, not allowing multiple loops, limiting continuous unpaired bases to 3, allowing no more 7 unpaired bases on a mature miRNA and not allowing any uncertain bases (“N”) in the pre-miRNA. Finally, 522 miRNA were identified as real miRNAs by testing their pre-miRNAs using our classification model.

In this study, we denoted the length of the sequence as *l*, the number of sequences as *n*, the length of the miRNA sequence as *k*, and the number of the miRNA sequences as *m*. Then, the average time complexity is *O*(*n* * *m* * *l* * *k*).

This time complexity was unacceptable. In this study, an algorithm was developed based on to the SEED algorithm [41] and the KMP algorithm. The known plant miRNA sequence was divided into four nearly equal sequence lengths. We compared these four sequences with the complete genome sequence of *Solanum lycopersicum* to identify similar fragments. When a matching pattern occurred, we completed the miRNA pairing with the corresponding positioning of the sequence. Sequences with less than 3 mismatches were saved. The average time complexity is *O*(4 * (*k*/4 + *l*) * *k* * *n* * *m*/4^*k*/4^).

Using the method described above, several potential miRNAs were obtained. Structural information regarding the miRNAs was obtained using RNAfold. Potential pre-miRNAs were obtained by identifying stem-loop-containing fragments, and potential pre-miRNAs with hairpins were obtained using the following criteria: stability above −4.42, %G + C content between 30% and 70%, less than 6 bases of mature miRNA that are not complementary with the other arm, no gaps in the complementary miRNA strand, no uncertain base (“N”) in the pre-miRNA, and less than 3 consecutive non-complementary bases.

As an important economic crop, *Solanum lycopersicum* is not only nutritious but also has various physiological functions that are relevant to the exploitation and development of plant resources. Currently, only 77 *Solanum lycopersicum* miRNAs are reported in miRbase, far less than the actual number of *Solanum lycopersicum* miRNAs. In this study, we found 522 *Solanum lycopersicum* miRNAs in the complete genome. Their sequence and ID in other plants are shown in Additional file 4.

**Figure 5:**
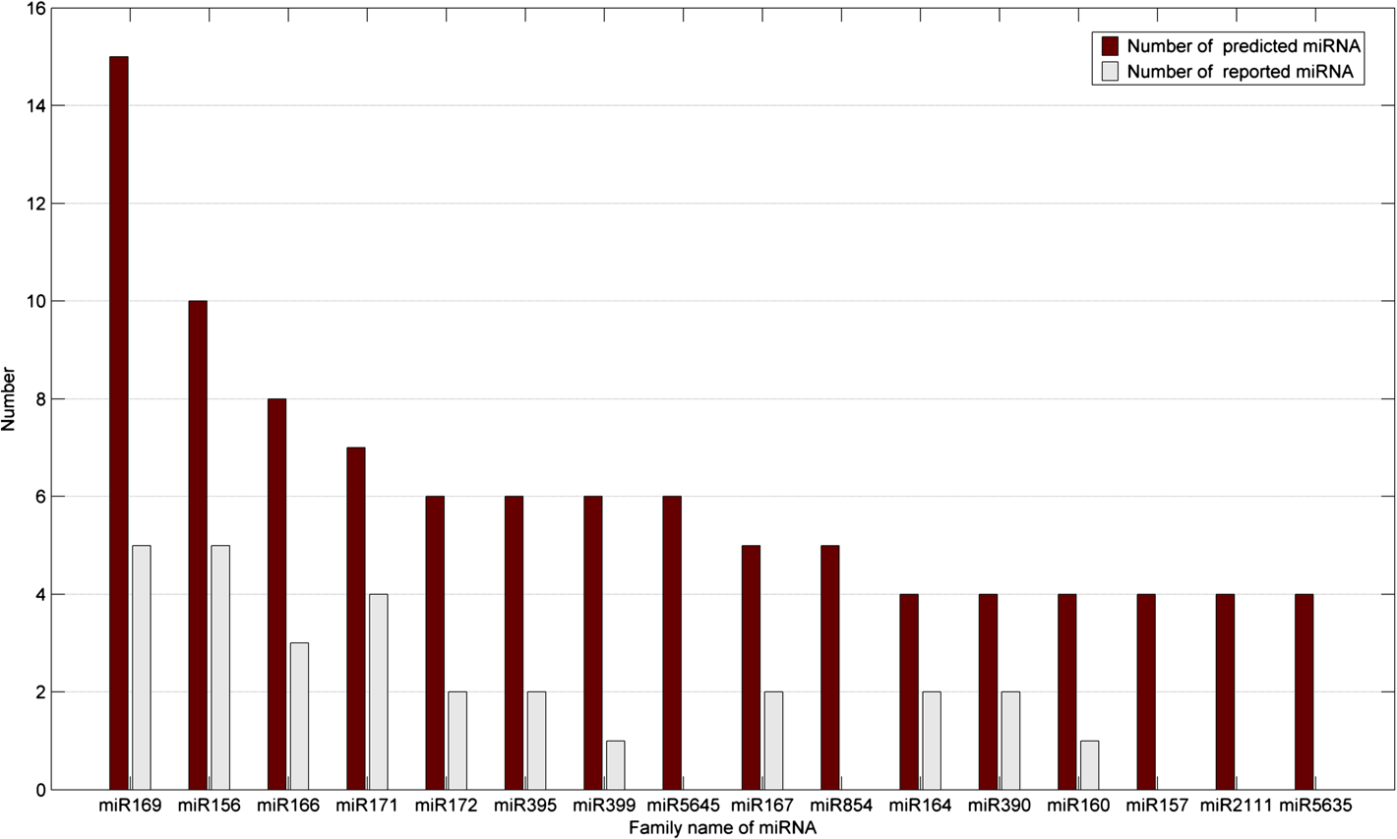
**Number of predicted members and reported number in** ***Solanum lycopersicumis.*** The number of predicted members which is more than 4 and the corresponding reported number in *Solanum lycopersicum*.

Figure 5 shows the number of predicted members which is more than 4 and the corresponding reported number in *Solanum lycopersicum*. (i) some of the predicted miRNAs have been reported as *Solanum lycopersicum* miRNAs in miRBase release 21.0; (ii) the more the members of miRMA family predicted in other plants, the more the members verified of this family in *Solanum lycopersicum*, it is concluded that their trends are similar with respect to the number of miRNA family; (iii) the number of miRNAs verified in *Solanum lycopersicum* is still less than the predicted number. Therefore, new miRNAs remain to be verified in the future.

## Discussion

In this study, a new classifier, miPlantPreMat, was developed for predicting plant pre-miRNAs and miRNAs. MiPlantPreMat was developed by analyzing existing miRNA prediction methods, combining the characteristics of plant pre-miRNAs, extracting features, selecting features and training samples to achieve efficient and effective classification. Importantly, 152 features were extracted to distinguish the hairpins of real/pseudo pre-miRNAs based on the characteristics of plant pre-miRNAs and miRNAs. After selecting the best subset for classification, 47 informative features were selected for use with miPlantPre, and 63 informative features were selected for use with miPlantMat. The accuracy, sensitivity and specificity of miPlantPreMat were all greater than 95% in terms of pre-miRNA classification and greater than 85% in terms of miRNA classification. Additionally, 522 potential miRNAs with stem-loop structures were found in the *Solanum lycopersicum* genome. The results of our study might prove useful for subsequent biological experiments.

## Conclusions

A comparison method was developed based on miRNA homology. Some miRNAs with low or specific expression patterns might not be found using this method. In the future, we intend to develop better classification models that can identify miRNAs with low and specific expression levels.

## Additional files

Additional file 1
**Feature extraction and selection of the SVM model.**
Additional file 2: Table S1 Ranking of the selected 47 features used in miPlantPre. Additional file 3: Table S2 Ranking of the selected 63 features used in miPlantMat. Additional file 4: Table S3 Sequence and ID in other plants of 522 predicted miRNAs.
