# Supplementary material for: Prediction of plant pre-microRNAs and their microRNAs in genome-scale sequences using structure-sequence features and support vector machine
Source: BMC Bioinformatics. 2014 Dec 30;15(1):423. doi: 10.1186/s12859-014-0423-x (PMC4310204; doi:10.1186/s12859-014-0423-x)
Supplement: Additional file 1 — Feature extraction and selection of the SVM model. [file 12859_2014_423_MOESM1_ESM.docx]

| Prediction of plant pre-microRNAs and their microRNAs in genome-scale sequences using [structure](app:ds:structure)-[sequence](app:ds:sequential) features and support vector machine  Jun Meng^1^,Dong Liu^1^, Chao Sun^1^, Yushi Luan^2,*^  ^1^School of Computer Science and Technology, Dalian University of Technology, Dalian, Liaoning 116023, China, ^2^School of Life Science and Biotechnology, Dalian University of Technology, Dalian, Liaoning 116023, China |
| --- |

# Feature SELECTION

This section explains the selection of 152 features in more detail.

## Features extracted in miPred

First we explain the 29 features which were previously used in *miPred* (Loong and Mishra, 2007) method.

1. **Sequential features**

These features were calculated from the primary RNA/DNA sequence. Let *L* be the length of a sequence.

- **16 dinucleotide frequencies**: $\%XY$where $X,Y\in\{A,C,G,U\}.$(%*AA*, %*AC*… %*UU*).

$\%XY=\left|XY\right|/(L-1)*100.$ **(1)**

Where$\left|XY\right|$denotes the number of dinucleotide XY in the sequence.

- **C+G content** [%(C+G)]

$\%(C+G)=(\left|C\right|+\left|G\right|)/L*100$ **(2)**

Where$\left|C\right|$ and $\left|G\right|$ respectively denote the number of nucleotide C and G in the sequence.

1. **Structural Features**

These structural features were calculated based on the RNA secondary structures predicted by *RNAfold* program (Hofacker, 2003) with the default parameters. *RNAfold* predicts the secondary structure having the minimum free energy (MFE) of folding from the primary sequence.

- **Normalized minimum free energy of folding (*dG*)**

$dG=(MFE/L)$ **(3)**

MFE can also be obtained from the *RNAfold* program with the predicted secondary structure. *dG* removes the bias that long sequences tend to have lower MFEs.

- **MFE Index 1 (*MFEI_1_*)**

$MFEI_{1}=dG/\%(C+G)$ **(4)**

- **MFE Index 2 (*MFEI_2_*)**

$MFEI_{2}=dG/n\_stems$ **(5)**

where *n_stems* is the number of stems in the secondary structure. A *stem* is a structural motif containing more than three contiguous base pairs.

- **Normalized base-paring propensity (*dP*)**

$dP=tot\_bases/L$ **(6)**

where *tot_bases* is the total number of base pairs in the secondary structure.

- **Normalized Shannon entropy (*dQ*) (Freyhult et al., 2005)**

In vivo, an RNA molecule commonly exists in an assembly of structures. The distribution of these structures can be modeled by a Boltzmann distribution of free energy. The probability of the structure$S_{\alpha}\in S(x)$is given by $P(s_{\alpha})=e^{-E_{\alpha}/RT}/Z$ where$Z=\sum_{S_{\alpha}\in S(x)} e^{-E_{\alpha}/RT}$,$E_{\alpha}$is the free energy of$S_{\alpha}$, $R=8.31451Jmol^{-1}K^{-1}$is the molar gas constant, and *T* is the temperature taken as 310.15K (37C).

The base pair probability $p_{ij}$(the probability that base *i* pair with base *j*) is then given by$p_{ij}=\sum_{S_{\alpha}\in S(x)} P(S_{\alpha})\delta_{ij}^{\alpha}$, where $\delta_{ij}^{\alpha}$is 1 if bases *i* and *j* is a base pair in$S_{\alpha}$, and 0 otherwise. The normalized Shannon entropy (*dQ*) of *x* is defined as

$dQ=\frac{-\sum_{i<j} p_{ij}.\log_{2}(p_{ij})}{L}$ **(7)**

- **Normalized base-pair distance (*dD*) (Freyhult et al., 2005)**

The base pair distance between two structures $S_{\alpha}$and $S_{\beta}$ on sequence *x* is defined as the number of base pairs not shared by the secondary structure $S_{\alpha}$ and$S_{\beta}$. The base pair distance between $S_{\alpha}$ and $S_{\beta}$ is equal to$d_{BP}(S_{\alpha},S_{\beta})=\sum_{i<j} (\delta_{ij}^{\alpha}+\delta_{ij}^{\beta}-2\delta_{ij}^{\alpha}\delta_{ij}^{\beta})$, where $\delta_{ij}^{\alpha}$is 1 if bases *i* and *j* is a base pair in$S_{\alpha}$, and 0 otherwise. The average base pair distance,$d_{BP}$, overall $S_{\alpha}$ and $S_{\beta}$structures can be defined as

$d_{BP}=\frac{1}{2}\sum_{S_{\alpha},S_{\beta}\in S(x)} [P(S_{\alpha})P(S_{\beta})\sum_{i<j} (\delta_{ij}^{\alpha}+\delta_{ij}^{\beta}-2\delta_{ij}^{\alpha}\delta_{ij}^{\beta})]=\sum_{i<j} (p_{ij}-p_{ij}^{2})$**(8)**

The simplification can be found in (Freyhult et al., 2005). Then the normalized base pair distance is given by

$dD=\frac{\sum_{i<j} (p_{ij}-p_{ij}^{2})}{L}$ **(9)**

- **The second (the Fielder) eigenvalue (*dF*)**

An RNA secondary structure *S* can be represented as a tree-graph *G*, where vertices represent loops, and edges represent stems. Laplacian matrix *L (G)* is a mathematical representation of a tree-graph *G*. The second eigenvalue (*dF*) of *L (G)* measures the compactness of a tree-graph. Therefore, *dF [L(S)] = dF [L (G)]* can be used as a similarity measure among a collection of RNA secondary structures.

- ***zG, zP. zQ, zD* and *zF***

In order to calculate the normalized variants (z values) for the structural features *dG, dP, dQ, dD* and *dF*, $R$number of random sequences were generated for each original sequence in the dataset by the 'Altschul-Erickson’ dinucleotide shuffling algorithm (Altschul and Erickson, 1985), which preserves both mono- and dinucleotide frequencies. When the random RNA sequences are generated the dinucleotide composition has to be preserved due to its relationship with the stacked base-pairs which is very important in the calculation of MFE (Workman and Krogh, 1999).

The z value for a feature dX of an original sequence is calculated as:

$Z(dX)=\frac{dX-\mu_{dX}}{\sigma_{dX}};\sigma_{dX}^{2}=\frac{1}{R-1}\sum_{i=1}^{R} (dX_{i}-\mu_{dX})^{2}$ **(10)**

Where $\mu_{dX}$and $\sigma_{dX}$are the sample mean and the standard deviation of the feature *dX* calculated for the *R* number of random sequences generated from the original sequences. The calculated z values for these features are represented using the variables *zG, zP, zQ, zD* and *zF*. We used *R=10^3^* as in this research.

All these 29 features were calculated using the scripts written for *miPred* research, which are available at <http://web.bii.a-star.edu.sg/~stanley/Publications/Supp_materials/06-002-supp.html>.

## microPred features

- 1. **New Minimum Free Energy (MFE)-related features**

**MFE Index 3 (*MEFI_3_*)**

*MFEI_3_ = dG/n _loops*  **(11)**

where *dG* is define in Eq. (3), and *n _loops* is the number of loops in the secondary structure.

- **MFE Index 4 (*MFEI_4_*)**

*MFEI_4_ = MFE/tot_bases*  **(12)**

where tot_bases is the total number of base pairs in the secondary structure.

- 1. **New RNAfold-related features**

As described earlier under the feature *dQ*, an RNA molecule commonly exists in assembly of structures and the distribution of these structures can be modeled by a Boltzmann distribution of free energy. The probability of the structure$S_{\alpha}\in S(x)$is given by $P(s_{\alpha})=e^{-E_{\alpha}/RT}/Z$ where$Z=\sum_{S_{\alpha}\in S(x)} e^{-E_{\alpha}/RT}$,$E_{\alpha}$is the free energy of$S_{\alpha}$, $R=8.31451Jmol^{-1}K^{-1}$is the molar gas constant, and T is the temperature taken as 310.15K (37C).

- **Normalized Ensemble Free Energy (*NEFE*) (Hofacker, 2003)**

$NEFE=EFE/L$ **(13)**

$$EFE=-RT\ln(Z)$$

- **The frequency of the MFE structure (*Freq*) (Hofacker, 2003)**

$Freq=e^{(EFE-MFE)/RT}$ **(14)**

- **The structural diversity (base pair distance) (*Diversity*)**

$Diversity=\sum_{i,j} p_{ij}(1-p_{ij})$ **(15)**

where $p_{ij}$is the probability of base i pair with base j. Basically, Diversity is the base pair distance described earlier under the feature dD, without being normalized by the sequence length L.

- **Related to these features we newly introduced the following feature:**

$Diff=|MFE-EFE|/L$ **(16)**

These features were extracted by the use of the *RNAfold* program with ‘*-p*’ option (under the default parameters at$37^{\circ}C$), which calculates the partition function and the base paring probability matrix following the algorithms presented in (McCaskill, 1990)

- 1. **New Mfold-related features**

The following thermodynamical features were calculated with the help of the *UNAfold* program <http://dinamelt.bioinfo.rpi.edu/twostate-fold.php> (Markham and Zuker, 2005) in the *Mfold* web server package (Zuker, 2003).

- **Structure Entropy (*dS)***
- **Normalized Structure Entropy (*dS/L)***
- **Structure Enthalpy (*dH)***
- **Normalized Structure Enthalpy (*dH/L)***
- **Melting Energy of the structure (*Tm*)**

*Tm* = *100* dH/dS* **(17)**

- **Normalized Melting Energy (*Tm/L)***

More details about these features can be found in (Markham and Zuker, 2005).

- 1. **New base pair-related features**

**Normalized base pair counts**

*|A-U|/L, |G-C|/L and |G-U|/L*

where *|X-Y|* is the number of (*X-Y)* base pairs in the secondary structure,$(X-Y)\in\{(A-U),(G-C),(G-U)\}$.

- **Average base pairs per stem**

*Avg_BP_Stem = tot_bases/n_stems* **(18)**

where *n_stems* is the number of stems in the secondary structure.

***%( A-U)/n_stems,* *%( G-C)/n_stems, %( G-U)/n_stems****.*

where %(X-Y) = |X-Y|/*tot_bases*e.

The additional scripts required to calculate the newly introduces features were written by us. All the scripts used to calculate these 48 features are available as a single package within the *microPred* program, which is available at <http://web.comlab.ox.ac.uk/people/ManoharaRukshan.Batuwita/microPred.htm>.

## PalntMiRNAPred features

**MFE Index 5 (*MEFI_5_*)**

*MFEI_5_= MFE/ %G+C_S* **(24)**

where %G+C_S is the GC content in the stems.

- **MFE Index 6 (*MFEI_6_*)**

*MFEI_6_ = MFE/* *stem_tot_bases* **(25)**

where *stem_tot_basesis* the number of base pairs in the stems.

- **Average number of mismatches per 21-nt window of mismatches per 21-nt window (*Avg_mis_num*) (Guo, 2011)**

*Avg_mis_num = tot_mismatches/n_21nts* **(26)**

where tot_mismatches is the total number of mismatches in the 21-nt sliding windows (which is roughly the length of a mature miRNA region and naturally has fewer than four successive mismatches) and n_21nts is the number of sliding windows in a stem.

## Triplet-SVM features

We exclude the terminal loop and external single-stranded regions of the hairpin and only consider the stem portions. The number of appearance of each triplet element is counted for each hairpin (pre-miRNA or pseudo pre-miRNA) to produce the 32-dimensional feature vector. It is normalized before being used as input features for SVM. (Xue, 2005)

## New features

Now we explain the 69 structural features introduced in miPlantPreMat, which have not been used for pre-miRNA classification problem before.

**MFE Index 7 (*MEFI_7_*)**

*MFEI_7_=MFE/%G+C_Begin_n_21nts* **(27)**

where *%G+C_Begin_n_21nts* is the GC content in the first 21 bases of the stems.

**MFE Index 8 (*MEFI_8_*)**

*MFEI_8_=MFE/%G+C_End_n_21nts* **(28)**

where *%G+C_End_n_21nts* is the GC content in the last 21 bases of the stems.

**MFE Index 9 (*MEFI_9_*)**

*MFEI_9_=MFE/avg_mis_num_n_21nts* **(29)**

where *avg_mis_num_n_21nts* is the average number of mismatches per 21-nt window.

- **The nucleotide unpaired with another nucleotide on the other side in the first 21 bases of the stems (*Mis_num_begin*).**
- **The nucleotide unpaired with another nucleotide on the other side in the last 21 bases of the stems (*Mis_num_end*).**
- **The triplet features as the frequ****encies of secondary structure extracted from the beginning and the ending of pre-miRNAs ("*G(((_begin_S*", "*A.(._end_S*", etc.).**

# SVM model selection AND IMPLEMENTATION

## SVM model selection

Interestingly, it has been found that the linear kernel could be seen as a special case of RBF and this relationship could be used to ease the parameter selection under RBF (Keerthi and Lin, 2003). In this method, first, a linear parameter search is conducted under the linear kernel and the optimal value for the parameter *C* is found. Let's call that value as$\tilde{C}$. Then, the range of one parameter (say$\gamma$) under the RBF is fixed. The corresponding best value of the other parameter (*C*) with respect to each value in the range of $\gamma$ can be calculated by Eq. (24). The derivation of this relationship is explained in (Keerthi and Lin, 2003).

$\log_{2}C=\log_{2}\tilde{C}-(1+\log_{2}\gamma)$ **(30)**

Under this method the parameter search of RBF becomes linear, which is much more efficient than the usual grid search, specially, with large datasets as ours. We used this method of model selection to train SVM models in this research. The performance of the classifier at each parameter point is evaluated by 5-fold cross-validation training on the training dataset using the *Gm* metric. Following the above method, we first considered the Linear kernel function and conducted a coarse parameter search with the value of $\log_{2}C=[-5,-4,...,20].$ Say we found the highest value for cross-validation *Gm* at $\log_{2}C=a.$ Then we conducted a narrow parameter search in the range $\log_{2}C=[a-0.75,a-0.5,...,a+0.75]$, found the optimal value for $\log_{2}C$, and fixed it as the value of $\log_{2}\tilde{C}.$ Then the RBF kernel was considered. We fixed the range$\log_{2}\gamma=[-20,-14,...5]$, and the corresponding values for $\log_{2}C$ for each value of $\log_{2}\gamma$was found by the Eq. (24). Then a coarse parameter search with each $(\log_{2}C,\log_{2}\gamma)$ was conducted. If we found the best value for cross-validation *Gm* at $\log_{2}\gamma=b$, again a narrow parameter search was conducted in the range $\log_{2}\gamma=[b-0.75,b-0.5,...,b+0.75]$ with the corresponding $\log_{2}C$ values found by Eq. (24). After finding the best parameter pair$(\log_{2}C,\log_{2}\gamma)$under the RBF, which gives the highest cross-validation *Gm* value for the training dataset, a new SVM model was trained using the complete training dataset at those parameters. This method is used to select the optimal parameters when developing all the SVM models in this research.

## Implementation details

The *matlab* interface of *libsvm2.86* (Chang and Lin, 2001) package was used to develop the SVM models in this research. All these experiments were programmed in *parallel matlab* and run in the ubuntu OS.
