# Supplementary material for: Prediction of plant pre-microRNAs and their microRNAs in genome-scale sequences using structure-sequence features and support vector machine
Source: BMC Bioinformatics. 2014 Dec 30;15(1):423. doi: 10.1186/s12859-014-0423-x (PMC4310204; doi:10.1186/s12859-014-0423-x)
Supplement: Additional file 2: Table S1 — Ranking of the selected 47 features used in miPlantPre. [file 12859_2014_423_MOESM2_ESM.docx]

| **Table S1 -Rranking of the selected 47 features using miPlantPre** | | | |
| --- | --- | --- | --- |
| No | Feature | IG | SVM-RFE Rank |
| 1 | dP | 0.78628 | 1 |
| 2 | MFEI5 | 0.77982 | 2 |
| 3 | zP | 0.75613 | 3 |
| 4 | A(((_S | 0.59366 | 46 |
| 5 | \|A-U\|/L | 0.56906 | 7 |
| 6 | zG | 0.54643 | 10 |
| 7 | dQ | 0.53468 | 31 |
| 8 | dS | 0.45531 | 9 |
| 9 | dH | 0.42444 | 4 |
| 10 | MFEI1 | 0.42196 | 40 |
| 11 | MFEI2 | 0.39259 | 22 |
| 12 | G..._S | 0.34812 | 14 |
| 13 | Tm | 0.343 | 6 |
| 14 | dD | 0.32999 | 36 |
| 15 | C..._S | 0.32066 | 24 |
| 16 | NEFE | 0.30957 | 19 |
| 17 | zF | 0.3091 | 11 |
| 18 | %AA | 0.26454 | 5 |
| 19 | %(A-U)/n_stems | 0.26277 | 45 |
| 20 | mis_num_begin | 0.23253 | 42 |
| 21 | G..(_S | 0.23074 | 17 |
| 22 | %UC | 0.22485 | 26 |
| 23 | MFEI3 | 0.21769 | 12 |
| 24 | %CA | 0.21749 | 39 |
| 25 | %G+C | 0.21311 | 29 |
| 26 | dG | 0.1974 | 32 |
| 27 | %GU | 0.18475 | 23 |
| 28 | %CG | 0.17212 | 20 |
| 29 | Diversity | 0.16729 | 33 |
| 30 | %AC | 0.13788 | 35 |
| 31 | Tm/L | 0.1333 | 21 |
| 32 | %GG | 0.12375 | 38 |
| 33 | MFEI6 | 0.1227 | 25 |
| 34 | %CU | 0.11651 | 8 |
| 35 | MFEI4 | 0.11603 | 15 |
| 36 | G..._S_end | 0.11563 | 34 |
| 37 | G(((_S | 0.07866 | 30 |
| 38 | C(((_S | 0.072 | 13 |
| 39 | %(G-C)/n_stems | 0.07079 | 44 |
| 40 | %GC | 0.06041 | 28 |
| 41 | %AU | 0.04384 | 27 |
| 42 | %CC | 0.03291 | 16 |
| 43 | Freq | 0.03099 | 37 |
| 44 | U..._S_end | 0.02868 | 47 |
| 45 | %GA | 0.02 | 18 |
| 46 | \|G-C\|/L | 0.01986 | 43 |
| 47 | A(.(_S_end | 0.00631 | 41 |
