# Supplementary material for: Prediction of plant pre-microRNAs and their microRNAs in genome-scale sequences using structure-sequence features and support vector machine
Source: BMC Bioinformatics. 2014 Dec 30;15(1):423. doi: 10.1186/s12859-014-0423-x (PMC4310204; doi:10.1186/s12859-014-0423-x)
Supplement: Additional file 3: Table S2 — Ranking of the selected 63 features used in miPlantMat. [file 12859_2014_423_MOESM3_ESM.docx]

| **Table S2 - Rranking of the selected 63 features using miPlantMat** | | | |
| --- | --- | --- | --- |
| No | Feature | IG | SVM-RFE rank |
| 1 | dP | 0.78628 | 1 |
| 2 | MFEI5 | 0.77982 | 2 |
| 3 | zP | 0.75613 | 3 |
| 4 | MFEI7 | 0.68656 | 54 |
| 5 | MFEI8 | 0.66704 | 48 |
| 6 | A(((_S | 0.59366 | 46 |
| 7 | MFEI9 | 0.56941 | 106 |
| 8 | \|A-U\|/L | 0.56906 | 7 |
| 9 | dQ | 0.53468 | 31 |
| 10 | avg_mis_num | 0.42474 | 113 |
| 11 | dH | 0.42444 | 4 |
| 12 | G..._S | 0.34812 | 14 |
| 13 | Tm | 0.343 | 6 |
| 14 | dD | 0.32999 | 36 |
| 15 | C..._S | 0.32066 | 24 |
| 16 | NEFE | 0.30957 | 19 |
| 17 | %AA | 0.26454 | 5 |
| 18 | %(A-U)/n_stems | 0.26277 | 45 |
| 19 | C..(_S | 0.24486 | 110 |
| 20 | mis_num_begin | 0.23253 | 42 |
| 21 | G..(_S | 0.23074 | 17 |
| 22 | %UC | 0.22485 | 26 |
| 23 | MFEI3 | 0.21769 | 12 |
| 24 | %CA | 0.21749 | 39 |
| 25 | mis_num_end | 0.21546 | 141 |
| 26 | U(((_S_end | 0.20861 | 122 |
| 27 | C((._S | 0.20476 | 142 |
| 28 | A(.._S | 0.19966 | 149 |
| 29 | C.((_S | 0.19026 | 130 |
| 30 | A(((_S_end | 0.17687 | 121 |
| 31 | Diversity | 0.16729 | 33 |
| 32 | G(.(_S | 0.14819 | 151 |
| 33 | G((._S | 0.14512 | 136 |
| 34 | Tm/L | 0.1333 | 21 |
| 35 | MFEI6 | 0.1227 | 25 |
| 36 | %CU | 0.11651 | 8 |
| 37 | MFEI4 | 0.11603 | 15 |
| 38 | U..._S | 0.09652 | 58 |
| 39 | G(((_S | 0.07866 | 30 |
| 40 | A..._S_end | 0.07662 | 74 |
| 41 | C(((_S | 0.072 | 13 |
| 42 | G..._S_begin | 0.06746 | 93 |
| 43 | G(((_S_begin | 0.04479 | 105 |
| 44 | %UG | 0.04059 | 89 |
| 45 | C(((_S_end | 0.03002 | 84 |
| 46 | %UA | 0.02957 | 111 |
| 47 | U..._S_end | 0.02868 | 47 |
| 48 | G(.._S_end | 0.02687 | 99 |
| 49 | A..(_S_begin | 0.0263 | 144 |
| 50 | %GA | 0.02 | 18 |
| 51 | \|G-C\|/L | 0.01986 | 43 |
| 52 | A(.._S_begin | 0.01862 | 49 |
| 53 | A(.._S_end | 0.0115 | 91 |
| 54 | G((._S_begin | 0.01047 | 69 |
| 55 | %UU | 0.01035 | 72 |
| 56 | C(.._S_end | 0.00879 | 95 |
| 57 | U.(._S_end | 0.00471 | 67 |
| 58 | G(((_S_end | 0.00359 | 104 |
| 59 | C((._S_end | 0.00339 | 146 |
| 60 | C(((_S_begin | 0.00205 | 85 |
| 61 | U..(_S_end | 0 | 145 |
| 62 | U(.._S_end | 0 | 131 |
| 63 | U.((_S_begin | 0 | 115 |
