# Supplementary material for: Prediction of plant pre-microRNAs and their microRNAs in genome-scale sequences using structure-sequence features and support vector machine
Source: BMC Bioinformatics. 2014 Dec 30;15(1):423. doi: 10.1186/s12859-014-0423-x (PMC4310204; doi:10.1186/s12859-014-0423-x)
Supplement: Additional file 4: Table S3 — Sequence and ID in other plants of 522 predicted miRNAs. [file 12859_2014_423_MOESM4_ESM.docx]

| **Table S3 -** **Sequence and ID of 522 predicted miRNAs** | | |
| --- | --- | --- |
| No. of miRNAs predicted | Sequence | ID of miRNAs in other plants |
| new miRNA 1 | UUCGCUUGCAGAGAGAAAUCAC | ath-miR173-5p |
| new miRNA 2 | CUGAAGUGUUUGGGGGAACUC | ath-miR395a |
| new miRNA 3 | UGAGAAGAAGAAGAAGAAAA | ath-miR5021 |
| new miRNA 4 | UUAGGCUAAGAUUUGUGAAGA | ath-miR1888b |
| new miRNA 5 | AUACCAAAACUCUCUCACUUU | ath-miR5638a |
| new miRNA 6 | UGAUUGGAAAUUUCGUUGACU | ath-miR779.2 |
| new miRNA 7 | UCUCGCGCUUGUACGGCUUU | ath-miR5642a |
| new miRNA 8 | UGGCAGGAAAGACAUAAUUUU | ath-miR5631 |
| new miRNA 9 | AUCCUCGGGAUACAGUUUACC | ath-miR2111b-3p |
| new miRNA 10 | UCCGGCAAGUUGACCUUGGCU | ath-miR169g-3p |
| new miRNA 11 | UGUGUUCUCAGGUCACCCCUG | ath-miR398c |
| new miRNA 12 | UUGGUGGACAAGAUCUGGGAU | ath-miR5665 |
| new miRNA 13 | ACACUUAGUUUUGUACAACAU | ath-miR5014a-5p |
| new miRNA 14 | UCAUUGAGUGCAUCGUUGAUG | ath-miR397b |
| new miRNA 15 | UCUUCUCCAAAUAGUUUAGGUU | ath-miR830-5p |
| new miRNA 16 | UCUAAGUCUUCUAUUGAUGUU | ath-miR776 |
| new miRNA 17 | AUGAUGAUGAUGAUGAUGAAA | ath-miR5658 |
| new miRNA 18 | UGGAAGAAGGUGAGACUUGCA | ath-miR5020a |
| new miRNA 19 | UCUUCACUAUUAGACGGACAA | ath-miR5999 |
| new miRNA 20 | UUUGUUAUUUUCGCAUGCUCC | ath-miR4239 |
| new miRNA 21 | UGAGCCUCUGUGGUAGCCCUCA | ath-miR771 |
| new miRNA 22 | UGGAAGAUGCUUUGGGAUUUAUU | ath-miR5654-3p |
| new miRNA 23 | CUGAAGUGUUUGGGGGAACUC | ath-miR395d |
| new miRNA 24 | UCGGACCAGGCUUCAUUCCCC | ath-miR166b |
| new miRNA 25 | AGGCUUUUAAGAUCUGGUUGC | ath-miR5643b |
| new miRNA 26 | UCACUCCUCUUCUUCUUGAUG | ath-miR847 |
| new miRNA 27 | UCAAUGCAUUGAAAGUGACUA | ath-miR161.2 |
| new miRNA 28 | GCUAAGAGCGGUUCUGAUGGA | ath-miR5630a |
| new miRNA 29 | UCGGACCAGGCUUCAUUCCCC | ath-miR166f |
| new miRNA 30 | UGGAAGAAGAUGAUAGAAUUA | ath-miR5641 |
| new miRNA 31 | UGGAUUGGUCAAGGGAAGCGU | ath-miR3440b-3p |
| new miRNA 32 | UUAUACCAAAUUAAUAGCAAA | ath-miR5017 |
| new miRNA 33 | GGUUCGUACGUACACUGUUCA | ath-miR416 |
| new miRNA 34 | ACAUAUGAUCUGCAUCUUUGC | ath-miR5595a |
| new miRNA 35 | CCGUAUCUUGGCCUUGUCAUU | ath-miR5024-3p |
| new miRNA 36 | UGAAGCUGCCAGCAUGAUCUGG | ath-miR167d |
| new miRNA 37 | UCAAUAGAUUGGACUAUGUAU | ath-miR860 |
| new miRNA 38 | UAACGCACAACACUAAGCCAU | ath-miR2939 |
| new miRNA 39 | UGGUAGCAGUAGCGGUGGUAA | ath-miR834 |
| new miRNA 40 | UCGGACCAGGCUUCAUUCCCC | ath-miR166a |
| new miRNA 41 | UAGUCCACUGUGGUCUAAGGC | ath-miR5639 |
| new miRNA 42 | UUAUGUCUUGUUGAUCUCAAU | ath-miR863-5p |
| new miRNA 43 | UGACAGAAGAGAGAGAGCAG | ath-miR156i |
| new miRNA 44 | CAGCCAAGGAUGACUUGCCGA | ath-miR169a |
| new miRNA 45 | ACAGUUUGUGUUUUGUUUUGU | ath-miR5998a |
| new miRNA 46 | UGUUUGUUGACAUCGGUCUAG | ath-miR833b |
| new miRNA 47 | ACAUAUGAUCUGCAUCUUUGC | ath-miR5995b |
| new miRNA 48 | UGCCAAAGGAGAUUUGCCCUG | ath-miR399a |
| new miRNA 49 | UUUAGGUCGAGCUUCAUUGGA | ath-miR843 |
| new miRNA 50 | UUGGGGACGACAUCUUUUGUUG | ath-miR447c-3p |
| new miRNA 51 | ACAGUUUGUGUUUUGUUUUGU | ath-miR5998b |
| new miRNA 52 | UUUGGAAAUAUUUGGCUUGACU | ath-miR5648-5p |
| new miRNA 53 | UUGACAGAAGAUAGAGAGCAC | ath-miR157b |
| new miRNA 54 | UAAUUUGGUGUUUCUUCGAUC | ath-miR870 |
| new miRNA 55 | UUAGAUGACCAUCAACAAACU | ath-miR827 |
| new miRNA 56 | UGAAGCUGCCAGCAUGAUCUA | ath-miR167b |
| new miRNA 57 | AUGAGUUGGGUCUAACCCAUAACU | ath-miR405d |
| new miRNA 58 | UUGAAGAGGACUUGGAACUUCGAU | ath-miR163 |
| new miRNA 59 | UGGGUGGUGAUCAUAUAAGAU | ath-miR823 |
| new miRNA 60 | UAUGGAAGAAAUUGUAGUAUU | ath-miR447a.2-3p |
| new miRNA 61 | CAGGUGGUUAGUGCAAUGGAA | ath-miR5660 |
| new miRNA 62 | UGCCUGGCUCCCUGUAUGCCA | ath-miR160a |
| new miRNA 63 | UUGGCAUUCUGUCCACCUCC | ath-miR394b |
| new miRNA 64 | UUGACAGAAGAUAGAGAGCAC | ath-miR157a |
| new miRNA 65 | GAUGAGGAUAGGGAGGAGGAG | ath-miR854d |
| new miRNA 66 | AUAAAUCCCAACAUCUUCCA | ath-miR5654-5p |
| new miRNA 67 | UUCGAGGCCUAUUAAACCUCUG | ath-miR402 |
| new miRNA 68 | UUAGGGUAGUUAACGGAAGUUA | ath-miR5629 |
| new miRNA 69 | UUGGACUGAAGGGAGCUCCCU | ath-miR319b |
| new miRNA 70 | UUUUACUGCUACUUGUGUUCC | ath-miR5012 |
| new miRNA 71 | UUGAUUCCCAAUCCAAGCAAG | ath-miR832-3p |
| new miRNA 72 | UUGGACUGAAGGGAGCUCCCU | ath-miR319a |
| new miRNA 73 | UGACAGAAGAGAGUGAGCAC | ath-miR156e |
| new miRNA 74 | AAUGAGAGAGAACACUGCAAA | ath-miR5029 |
| new miRNA 75 | UGCCAAAGGAGAGUUGCCCUG | ath-miR399b |
| new miRNA 76 | UUGAGCCGUGCCAAUAUCACG | ath-miR171c |
| new miRNA 77 | UAGCCAAGGAUGACUUGCCUG | ath-miR169h |
| new miRNA 78 | UUGAAUUGAAGUGCUUGAAUU | ath-miR846 |
| new miRNA 79 | UUAGAGUUUUCUGGAUACUUA | ath-miR781b |
| new miRNA 80 | UAACUAUUUUGAGAAGAAGUG | ath-miR830-3p |
| new miRNA 81 | UAAUCUGCAUCCUGAGGUUUA | ath-miR2111a-5p |
| new miRNA 82 | AGAAGCAAAAUGACGACUCGG | ath-miR3933 |
| new miRNA 83 | UCUCUCUGUUGUGAAGUCAAA | ath-miR859 |
| new miRNA 84 | UCAUGGUCAGAUCCGUCAUCC | ath-miR842 |
| new miRNA 85 | UGACUAGACCCGUAACAUUAC | ath-miR4240 |
| new miRNA 86 | AUAGUCAAUUUUAUCGGUCUG | ath-miR5664 |
| new miRNA 87 | UCUCGGUUCGCGAUCCACAAG | ath-miR851-5p |
| new miRNA 88 | UGAAGCUGCCAGCAUGAUCUA | ath-miR167a |
| new miRNA 89 | GCAGCACCAUUAAGAUUCAC | ath-miR172b-5p |
| new miRNA 90 | AGGCUUUUAAGAUCUGGUUGC | ath-miR5643a |
| new miRNA 91 | CUUGAGAGAGAGAACACAGACG | ath-miR2936 |
| new miRNA 92 | UGUUAAGGAGUGUUAACGGUG | ath-miR5635d |
| new miRNA 93 | AUGAGUUGGGUCUAACCCAUAACU | ath-miR405a |
| new miRNA 94 | AGCUCUGAUACCAAAUGAUGGAAU | ath-miR829.1 |
| new miRNA 95 | UAGCCAAGGAUGACUUGCCUG | ath-miR169k |
| new miRNA 96 | GAUCUUUUGAGAGGGUUCCAG | ath-miR2938 |
| new miRNA 97 | GCUAAGAGCGGUUCUGAUGGA | ath-miR5630b |
| new miRNA 98 | UUCUGCUAUGUUGCUGCUCAU | ath-miR779.1 |
| new miRNA 99 | UAGAAUGCUAUUGUAAUCCAG | ath-miR406 |
| new miRNA 100 | UUCUUGUGGAUUCCUUGGAAA | ath-miR5016 |
| new miRNA 101 | UUCGAUGUCUAGCAGUGCCA | ath-miR775 |
| new miRNA 102 | UUUUUCCUCAAAUUUAUCCAA | ath-miR865-3p |
| new miRNA 103 | ACUGUAUAUAUGUAAGUGACA | ath-miR5025 |
| new miRNA 104 | UGUUAAGGAGUGUUAACGGUG | ath-miR5635a |
| new miRNA 105 | AAGAUAAGCGCCUUAGUUCUG | ath-miR852 |
| new miRNA 106 | AAACGAACAAAAAACUGAUGG | ath-miR837-3p |
| new miRNA 107 | UCAGGUAUGAUUGACUUCAAA | ath-miR864-5p |
| new miRNA 108 | UUAUGAAUGCUGAGGAUGUUG | ath-miR419 |
| new miRNA 109 | GGCAAUAACUUGAGCAAACA | ath-miR773b-5p |
| new miRNA 110 | GGAAUCUUGAUGAUGCUGCAU | ath-miR172e |
| new miRNA 111 | UUCCACAGCUUUCUUGAACUG | ath-miR396a |
| new miRNA 112 | UCGGACCAGGCUUCAUUCCCC | ath-miR166g |
| new miRNA 113 | AUCUGAAGAAAAUAGCGGCAU | ath-miR5648-3p |
| new miRNA 114 | CAUCCAAGGUGUUUGUAGAAA | ath-miR2934-3p |
| new miRNA 115 | UUUGGAUUGAAGGGAGCUCUU | ath-miR159b |
| new miRNA 116 | UCGGACCAGGCUUCAUUCCCC | ath-miR166c |
| new miRNA 117 | UUGGCAUUCUGUCCACCUCC | ath-miR394a |
| new miRNA 118 | UUGGUUACCCAUAUGGCCAUC | ath-miR774a |
| new miRNA 119 | UGACAGAAGAGAGAGAGCAC | ath-miR156j |
| new miRNA 120 | CUGAAGUGUUUGGGGGAACUC | ath-miR395e |
| new miRNA 121 | UCUCGCGCUUGUACGGCUUU | ath-miR5642b |
| new miRNA 122 | UCGAUAAACCUCUGCAUCCAG | ath-miR162b |
| new miRNA 123 | UAAGCUGCCAGCAUGAUCUUG | ath-miR167c |
| new miRNA 124 | UCCUGUGUUUCCUUUGAUGCGUGG | ath-miR836 |
| new miRNA 125 | AACCUAUUUAACGACAUGACU | ath-miR5645c |
| new miRNA 126 | UGAGAGAAGUGAGAUGAAAUC | ath-miR1886.1 |
| new miRNA 127 | UGGAGAAGCAGGGCACGUGCG | ath-miR164c |
| new miRNA 128 | UUGACAGAAGAUAGAGAGCAC | ath-miR157c |
| new miRNA 129 | UGUUGGGAAAGAAAAACUCUU | ath-miR5019 |
| new miRNA 130 | UCGCUUGGUGCAGGUCGGGAA | ath-miR168b |
| new miRNA 131 | GAUGAGGAUAGGGAGGAGGAG | ath-miR854e |
| new miRNA 132 | CAUCCAUAUUUUCAUCUCGAA | ath-miR774b-3p |
| new miRNA 133 | UAGCCAAGGAUGACUUGCCUG | ath-miR169l |
| new miRNA 134 | UUUUGGAAAUUUGUCCUUACG | ath-miR426 |
| new miRNA 135 | UUGGUGUUAUGUGUAGUCUUC | ath-miR5015 |
| new miRNA 136 | UAGACCAUUUGUGAGAAGGGA | ath-miR824 |
| new miRNA 137 | UUGGAUUUAUAGUUGGAUAAG | ath-miR5632 |
| new miRNA 138 | AACUUUGUGAUGACAACGAAG | ath-miR3932b |
| new miRNA 139 | GUCAUGGGGUAUGAUCGAAUG | ath-miR5022 |
| new miRNA 140 | UGAAAGUGACUACAUCGGGGU | ath-miR161.1 |
| new miRNA 141 | AGAAUCUUGAUGAUGCUGCAU | ath-miR172a |
| new miRNA 142 | AGAAUCUUGAUGAUGCUGCAG | ath-miR172d |
| new miRNA 143 | UUUUCCUCUGUUGAAUUCUUGC | ath-miR4221 |
| new miRNA 144 | UCGCUCUGAUACCAAAUUGAUG | ath-miR845b |
| new miRNA 145 | UCGGACCAGGCUUCAUCCCCC | ath-miR165b |
| new miRNA 146 | AAGCUCAGGAGGGAUAGCGCC | ath-miR390b |
| new miRNA 147 | AACAGAGCAGAAACAGAACAU | ath-miR415 |
| new miRNA 148 | UUGUUUUGGAUCUUAGAUACA | ath-miR5650 |
| new miRNA 149 | UACUAAGUAGAGUCUAAGAGA | ath-miR1887 |
| new miRNA 150 | CGUAGUUGCAGAGCUUGACGG | ath-miR5636 |
| new miRNA 151 | UAAAGUCAAUAAUACCUUGAAG | ath-miR864-3p |
| new miRNA 152 | UGACAGAAGAGAGUGAGCAC | ath-miR156f |
| new miRNA 153 | UGCCAAAGGAGAGUUGCCCUG | ath-miR399c |
| new miRNA 154 | CUUUAUAUCCGCAUUUGCGCA | ath-miR2112-3p |
| new miRNA 155 | UUUGGAUUGAAGGGAGCUCCU | ath-miR159c |
| new miRNA 156 | ACAAACACCUUGGAUGUUCUU | ath-miR782 |
| new miRNA 157 | UUUAAAUCAUAUACUUUUGGU | ath-miR407 |
| new miRNA 158 | UAAUGUGAUGAUGAACUGACC | ath-miR418 |
| new miRNA 159 | UGCGGGAAGCAUUUGCACAUG | ath-miR822 |
| new miRNA 160 | CAGCCAAGGAUGACUUGCCGG | ath-miR169c |
| new miRNA 161 | UUGAGAGCAACAAGACAUAAU | ath-miR863-3p |
| new miRNA 162 | UUUUCUUGGCCCAUCCACUUC | ath-miR3440b-5p |
| new miRNA 163 | AUUGGUUCAAUUCUGGUGUUG | ath-miR869.1 |
| new miRNA 164 | AGCUAAGGAUUUGCAUUCUCA | ath-miR5663 |
| new miRNA 165 | UCGGACCAGGCUUCAUCCCCC | ath-miR165a |
| new miRNA 166 | GUGGGUUGCGGAUAACGGUA | ath-miR5644 |
| new miRNA 167 | AUGGCAUGAAAGAAGGUGAGA | ath-miR5020b |
| new miRNA 168 | UGCUGGGAUCGGGAAUCGAAA | ath-miR832-5p |
| new miRNA 169 | UUUUCUUCUACUUCUUGCACA | ath-miR838 |
| new miRNA 170 | GAUGAGGAUAGGGAGGAGGAG | ath-miR854b |
| new miRNA 171 | UUAGAGUUUUCUGGAUACUUA | ath-miR781a |
| new miRNA 172 | UGGAGAAGAUACGCAAGAAAG | ath-miR835-3p |
| new miRNA 173 | CGACAGAAGAGAGUGAGCAC | ath-miR156g |
| new miRNA 174 | UUAGAUUCACGCACAAACUCG | ath-miR403 |
| new miRNA 175 | UCAUCUUCAUCAUCAUCGUCA | ath-miR414 |
| new miRNA 176 | AUGAGUUGGGUCUAACCCAUAACU | ath-miR405b |
| new miRNA 177 | UACGAGCCACUUGAAACUGAA | ath-miR841a |
| new miRNA 178 | GUUCGAGGCACGUUGGGAGG | ath-miR5646 |
| new miRNA 179 | AUGAAUUUGGAUCUAAUUGAG | ath-miR865-5p |
| new miRNA 180 | UUCCACAGCUUUCUUGAACUU | ath-miR396b |
| new miRNA 181 | ACCGGUUGGAACUUGCCUUAA | ath-miR5027 |
| new miRNA 182 | UGGCUUGGUUUAUGUACACCG | ath-miR778 |
| new miRNA 183 | AUAAGAGCUGUUGAAGGAGUC | ath-miR2937 |
| new miRNA 184 | UCGGACCAGGCUUCAUUCCCC | ath-miR166d |
| new miRNA 185 | GUCCUCGGGAUGCGGAUUACC | ath-miR2111a-3p |
| new miRNA 186 | UGACAGAAGAGAGUGAGCAC | ath-miR156d |
| new miRNA 187 | UUGUGCGGUUCAAAUAGUAAC | ath-miR5651 |
| new miRNA 188 | CUGAAGUGUUUGGGGGGACUC | ath-miR395f |
| new miRNA 189 | UGCCUGGCUCCCUGUAUGCCA | ath-miR160c |
| new miRNA 190 | UUUGCUUCCAGCUUUUGUCUC | ath-miR773a |
| new miRNA 191 | CCCCAAAUGUAGACAAAGCA | ath-miR158b |
| new miRNA 192 | UGUUAAGGAGUGUUAACGGUG | ath-miR5635b |
| new miRNA 193 | UCAAGGAACGGAUUUUGUUAA | ath-miR866-5p |
| new miRNA 194 | AAUGCGCAACUCUAUAUUUCC | ath-miR5637 |
| new miRNA 195 | UCCCCUCUUUAGCUUGGAGAAG | ath-miR853 |
| new miRNA 196 | UGAGAUGAAAUCUUUGAUUGG | ath-miR1886.2 |
| new miRNA 197 | UAUGAUCAUCAGAAAACAGUG | ath-miR5633 |
| new miRNA 198 | AUAUGCUGGAUCUACUUGAAG | ath-miR862-3p |
| new miRNA 199 | UAUGAGAGUAUUAUAAGUCAC | ath-miR400 |
| new miRNA 200 | ACAAAAUCCGUCUUUGAAGA | ath-miR866-3p |
| new miRNA 201 | UUGAAUGUGAAUGAAUCGGGC | ath-miR5652 |
| new miRNA 202 | UGAGCCAAGGAUGACUUGCCG | ath-miR169g-5p |
| new miRNA 203 | AGAAUCUUGAUGAUGCUGCAG | ath-miR172c |
| new miRNA 204 | UGCCAAAGGAGAUUUGCCCGG | ath-miR399f |
| new miRNA 205 | AUUUGAGUCAUGUCGUUAAG | ath-miR5645b |
| new miRNA 206 | UCAUUGAGUGCAGCGUUGAUG | ath-miR397a |
| new miRNA 207 | ACUCAUAAGAUCGUGACACGU | ath-miR5026 |
| new miRNA 208 | UUUGGAUUGAAGGGAGCUCUA | ath-miR159a |
| new miRNA 209 | UGAGCCAAGGAUGACUUGCCG | ath-miR169d |
| new miRNA 210 | AUUUGAGUCAUGUCGUUAAG | ath-miR5645f |
| new miRNA 211 | UCCAAUAGGUCGAGCAUGUGC | ath-miR862-5p |
| new miRNA 212 | CUGAAGUGUUUGGGGGGACUC | ath-miR395c |
| new miRNA 213 | ACAAAGUUUUAUACUGACAAU | ath-miR4245 |
| new miRNA 214 | UGAUUCUCUGUGUAAGCGAAA | ath-miR173-3p |
| new miRNA 215 | GAUGAGGAUAGGGAGGAGGAG | ath-miR854a |
| new miRNA 216 | UAAGUUAAGAUUUGUGAAGAA | ath-miR1888a |
| new miRNA 217 | UAGCCAAGGAUGACUUGCCUG | ath-miR169m |
| new miRNA 218 | UUGAAAUUGUAGAUUUCGUAC | ath-miR4243 |
| new miRNA 219 | AUUGGUAGUGGAUAAGGGGGC | ath-miR5023 |
| new miRNA 220 | UACGCAUUGAGUUUCGUUGCUU | ath-miR777 |
| new miRNA 221 | AGCAAAAGCUAAGGAAAAGGAA | ath-miR855 |
| new miRNA 222 | AAGUAGACACAUAAGAAGGAG | ath-miR5655 |
| new miRNA 223 | UGCCAAAGGAGAUUUGCCCCG | ath-miR399d |
| new miRNA 224 | UUAAAGCUCCACCAUGAGUCCAAU | ath-miR5018 |
| new miRNA 225 | UCUAGCAGCUGUUGAGCAGGU | ath-miR780.1 |
| new miRNA 226 | UCAUGUCGUAAUAGUAGUCAC | ath-miR868-5p |
| new miRNA 227 | UCAGAGUAUCAGCCAUGUGA | ath-miR3434-3p |
| new miRNA 228 | ACUGAAGUAGAGAUUGGGUUU | ath-miR5656 |
| new miRNA 229 | UGAUUGAGCCGCGCCAAUAUC | ath-miR171a |
| new miRNA 230 | UCUUUCUGCAAACGCCUUGGA | ath-miR2934-5p |
| new miRNA 231 | UCCAAAGGGAUCGCAUUGAUCC | ath-miR393a |
| new miRNA 232 | UAGUCCGGUUUUGGAUACGUG | ath-miR826 |
| new miRNA 233 | UGACAGAAGAUAGAGAGCAC | ath-miR157d |
| new miRNA 234 | UGUGUUCUCAGGUCACCCCUU | ath-miR398a |
| new miRNA 235 | AUGGGACAUCGAGCAUUUAAU | ath-miR5666 |
| new miRNA 236 | AUAGUUUCUCUUGUUCUGCAC | ath-miR413 |
| new miRNA 237 | UUGGGGACGAGAUGUUUUGUUG | ath-miR447b |
| new miRNA 238 | GAAAUCGGAGAGGAAAUUCGCC | ath-miR2933b |
| new miRNA 239 | AUUUGUACACCUAGAUCUGUA | ath-miR5014b |
| new miRNA 240 | UGAAACCAAGUAGCUAAAUAG | ath-miR5997 |
| new miRNA 241 | UGACAGAAGAGAGUGAGCAC | ath-miR156a |
| new miRNA 242 | UCUGGUGUUGAGAUAGUUGAC | ath-miR869.2 |
| new miRNA 243 | CUUCUUAAGUGCUGAUAAUGC | ath-miR868-3p |
| new miRNA 244 | AACUUUGUGAUGACAACGAAG | ath-miR3932a |
| new miRNA 245 | UUCGUUGUCUGUUCGACCUUG | ath-miR858b |
| new miRNA 246 | UCAAGUUUGAUGACGAUUCCA | ath-miR5647 |
| new miRNA 247 | UCCAAAGGGAUCGCAUUGAUCC | ath-miR393b |
| new miRNA 248 | CAAAUUAAAGCUUCAAGGUAG | ath-miR829.2 |
| new miRNA 249 | ACUUGGCUGAUUCUAUUAUU | ath-miR3434-5p |
| new miRNA 250 | AGAGGUGACCAUUGGAGAUG | ath-miR5662 |
| new miRNA 251 | CAAUUUCUAGUGGGUCGUAUU | ath-miR841b-3p |
| new miRNA 252 | ACAGUGGUCAUCUGGUGGGCU | ath-miR5638b |
| new miRNA 253 | UGACAGAAGAAAGAGAGCAC | ath-miR156h |
| new miRNA 254 | CAGCCAAGGAUGACUUGCCGG | ath-miR169b |
| new miRNA 255 | GAUGGAUAUGUCUUCAAGGAC | ath-miR861-3p |
| new miRNA 256 | AGGGACUUUGUGAAUUUAGGG | ath-miR5634 |
| new miRNA 257 | CGGCUCUGAUACCAAUUGAUG | ath-miR845a |
| new miRNA 258 | UGGGUUGAGUUGAGUUGAGUUGGC | ath-miR5653 |
| new miRNA 259 | AUUUGAGUCAUGUCGUUAAG | ath-miR5645d |
| new miRNA 260 | UACGAGCCACUGGAAACUGAA | ath-miR841b-5p |
| new miRNA 261 | UAAGAUCCGGACUACAACAAAG | ath-miR850 |
| new miRNA 262 | AAGCUUUGCUCGUUCAUGUUC | ath-miR783 |
| new miRNA 263 | UAGCCAAGGAUGACUUGCCUG | ath-miR169n |
| new miRNA 264 | CGAAACUGGUGUCGACCGACA | ath-miR401 |
| new miRNA 265 | UCGCUUGGUGCAGGUCGGGAA | ath-miR168a |
| new miRNA 266 | UCGAUAAACCUCUGCAUCCAG | ath-miR162a |
| new miRNA 267 | CCUUGGAGAAAUAUGCGUCAA | ath-miR861-5p |
| new miRNA 268 | AUUGAAUAUGUUGGUUACUAU | ath-miR5649a |
| new miRNA 269 | UUGGGGACGAGAUGUUUUGUUG | ath-miR447a-3p |
| new miRNA 270 | UAGCCAAGGAUGACUUGCCUG | ath-miR169i |
| new miRNA 271 | UGGACAAGGUUAGAUUUGGUG | ath-miR5657 |
| new miRNA 272 | AAGCUCAGGAGGGAUAGCGCC | ath-miR390a |
| new miRNA 273 | GAAAUAGCGAAGAUAUGAUUA | ath-miR5628 |
| new miRNA 274 | UACCAACCUUUCAUCGUUCCC | ath-miR839 |
| new miRNA 275 | AUAGCCUUGAACGCCGUCGUU | ath-miR4228 |
| new miRNA 276 | UUUCGUUGUCUGUUCGACCUU | ath-miR858a |
| new miRNA 277 | CGAUGAAGGUCUUUGGAACGGUA | ath-miR5659 |
| new miRNA 278 | UUCUUCGUGAAUAUCUGGCAU | ath-miR780.2 |
| new miRNA 279 | UAGACCGAUGUCAACAAACAAG | ath-miR833a-3p |
| new miRNA 280 | CGCAAAUGCGGAUAUCAAUGU | ath-miR2112-5p |
| new miRNA 281 | GAAGGUAGUGAAUUUGUUCGA | ath-miR417 |
| new miRNA 282 | UUUUGUAUGUUGAAGGUGUAU | ath-miR857 |
| new miRNA 283 | UGGCAUGGAAGAAGGUGAGAC | ath-miR5020c |
| new miRNA 284 | UUCGCAGGAGAGAUAGCGCCA | ath-miR391 |
| new miRNA 285 | UAAACUAAUCACGGAAAUGCA | ath-miR420 |
| new miRNA 286 | CUGAAGUGUUUGGGGGGACUC | ath-miR395b |
| new miRNA 287 | UGGAGAAGCAGGGCACGUGCA | ath-miR164b |
| new miRNA 288 | UGACAGAAGAGAGUGAGCAC | ath-miR156c |
| new miRNA 289 | UUUGAUUCCAGCUUUUGUCUC | ath-miR773b-3p |
| new miRNA 290 | AAUUAAAGAUUUCAUCUUACU | ath-miR1886.3 |
| new miRNA 291 | UAAUCUGCAUCCUGAGGUUUA | ath-miR2111b-5p |
| new miRNA 292 | UGGUAAGAUUGCUUAUAAGCU | ath-miR844-5p |
| new miRNA 293 | GAUGAGGAUAGGGAGGAGGAG | ath-miR854c |
| new miRNA 294 | UGAGCCAAGGAUGACUUGCCG | ath-miR169e |
| new miRNA 295 | UUGUACAAAUUUAAGUGUACG | ath-miR5014a-3p |
| new miRNA 296 | UAAUCCUACCAAUAACUUCAGC | ath-miR856 |
| new miRNA 297 | UGACAUCCAGAUAGAAGCUUUG | ath-miR5996 |
| new miRNA 298 | UGACAUGGGACUGCCUAAGCUA | ath-miR848 |
| new miRNA 299 | AUGCACUGCCUCUUCCCUGGC | ath-miR408 |
| new miRNA 300 | UGAGAGAAGGAAUUAGAUUCA | ath-miR5640 |
| new miRNA 301 | UGAUCUCUUCGUACUCUUCUUG | ath-miR831 |
| new miRNA 302 | UUCUCAAGAAGGUGCAUGAAC | ath-miR825 |
| new miRNA 303 | UCUUGCUUAAAUGAGUAUUCCA | ath-miR828 |
| new miRNA 304 | AUGACAAGGCCAAGAUAUAACA | ath-miR5024-5p |
| new miRNA 305 | CCCCUUACAAUGUCGAGUAAA | ath-miR447c-5p |
| new miRNA 306 | UGCCAAAGGAGAUUUGCCUCG | ath-miR399e |
| new miRNA 307 | UCACUGGUACCAAUCAUUCCA | ath-miR4227 |
| new miRNA 308 | UGCCUGGCUCCCUGUAUGCCA | ath-miR160b |
| new miRNA 309 | UGUUUGUUGUACUCGGUCUAGU | ath-miR833a-5p |
| new miRNA 310 | ACACUGAAGGACCUAAACUAAC | ath-miR840 |
| new miRNA 311 | GAAAUCGGAGAGGAAAUUCGCC | ath-miR2933a |
| new miRNA 312 | AGAAUCUUGAUGAUGCUGCAU | ath-miR172b-3p |
| new miRNA 313 | UGGAGAAGCAGGGCACGUGCA | ath-miR164a |
| new miRNA 314 | UGAUUGAGCCGUGUCAAUAUC | ath-miR170 |
| new miRNA 315 | UGGGUGGCAAACAAAGACGAC | ath-miR851-3p |
| new miRNA 316 | UCGGACCAGGCUUCAUUCCCC | ath-miR166e |
| new miRNA 317 | AUCAGUUUCUUGUUCGUUUCA | ath-miR837-5p |
| new miRNA 318 | UUAUAAGCCAUCUUACUAGUU | ath-miR844-3p |
| new miRNA 319 | AAUUGGGUUUAUGCUAGAGUU | ath-miR5028 |
| new miRNA 320 | UCCCAAAUGUAGACAAAGCA | ath-miR158a |
| new miRNA 321 | UAGCCAAGGAUGACUUGCCUG | ath-miR169j |
| new miRNA 322 | UUGGACUGAAGGGAGCUCCUU | ath-miR319c |
| new miRNA 323 | UGACAGAAGAGAGUGAGCAC | ath-miR156b |
| new miRNA 324 | UUCUUGCAUAUGUUCUUUAUC | ath-miR835-5p |
| new miRNA 325 | AUUUGAGUCAUGUCGUUAAG | ath-miR5645e |
| new miRNA 326 | AUUGAAUAUGUUGGUUACUAU | ath-miR5649b |
| new miRNA 327 | UUGAGCCGUGCCAAUAUCACG | ath-miR171b |
| new miRNA 328 | UUUGUGACAUCUAGGUGCUUU | ath-miR5013 |
| new miRNA 329 | AUUAACGCUGGCGGUUGCGGCAGC | ath-miR404 |
| new miRNA 330 | UGAGAUGAAGAUAUGGGUGAU | ath-miR774b-5p |
| new miRNA 331 | UGUGUUCUCAGGUCACCCCUG | ath-miR398b |
| new miRNA 332 | AGAGGUACAUCAUGUAGUCUG | ath-miR5661 |
| new miRNA 333 | UAACUAAACAUUGGUGUAGUA | ath-miR849 |
| new miRNA 334 | UUGAACAUGGUUUAUUAGGAA | ath-miR867 |
| new miRNA 335 | UGUUAAGGAGUGUUAACGGUG | ath-miR5635c |
| new miRNA 336 | UUUUUCCUACUCCGCCCAUACC | ath-miR472 |
| new miRNA 337 | AUUUGAGUCAUGUCGUUAAG | ath-miR5645a |
| new miRNA 338 | UGAGCCAAGGAUGACUUGCCG | ath-miR169f |
| new miRNA 339 | AAGCUCAGGAGGGAUAGCGCC | bna-miR390a |
| new miRNA 340 | UGCCUGGCUCCCUGUAUGCCA | bna-miR160b |
| new miRNA 341 | UCCAAAGGGAUCGCAUUGAUC | bna-miR393 |
| new miRNA 342 | UAGCCAAGGAUGACUUGCCUGC | bna-miR169l |
| new miRNA 343 | UCAUUGAGUGCAGCGUUGAUGU | bna-miR397b |
| new miRNA 344 | UGAAGCUGCCAGCAUGAUCUAA | bna-miR167b |
| new miRNA 345 | UCCACCCAUACCAUACAGACCC | bna-miR6030 |
| new miRNA 346 | UAGACCAUUUGUGAGAAGGGA | bna-miR824 |
| new miRNA 347 | UCGGACCAGGCUUCAUUCCCC | bna-miR166d |
| new miRNA 348 | AAGCUCAGGAGGGAUAGCGCC | bna-miR390b |
| new miRNA 349 | UUGACAGAAGAUAGAGAGCAC | bna-miR156g |
| new miRNA 350 | UCGCUUGGUGCAGGUCGAGAA | bna-miR168b |
| new miRNA 351 | UGGAGAAGCAGGGCACGUGCA | bna-miR164a |
| new miRNA 352 | AGAAUCUUGAUGAUGCUGCAU | bna-miR172a |
| new miRNA 353 | UCGGACCAGGCUUCAUUCCCC | bna-miR166e |
| new miRNA 354 | UGGAGAAGCAGGGCACGUGCG | bna-miR164b |
| new miRNA 355 | ACAGCCUAAACCAAUCGGAGC | bna-miR1140 |
| new miRNA 356 | UAGCCAAGGAUGACUUGCCUGC | bna-miR169k |
| new miRNA 357 | UCAAUGCACUGAAAGUGACUA | bna-miR161 |
| new miRNA 358 | UUGAGCCGUGCCAAUAUCACG | bna-miR171a |
| new miRNA 359 | CUGAAGUGUUUGGGGGAACUC | bna-miR395b |
| new miRNA 360 | UGAAGCUGCCAGCAUGAUCUAA | bna-miR167a |
| new miRNA 361 | AUCCUCGGGAUACAGAUUACC | bna-miR2111b-3p |
| new miRNA 362 | CAGCCAAGGAUGACUUGCCGA | bna-miR169a |
| new miRNA 363 | UGCCUGGCUCCCUGUAUGCCA | bna-miR160c |
| new miRNA 364 | UCGGACCAGGCUUCAUUCCCC | bna-miR166b |
| new miRNA 365 | UGACAGAAGAGAGUGAGCAC | bna-miR156d |
| new miRNA 366 | UAGCCAAGGAUGACUUGCCUGC | bna-miR169g |
| new miRNA 367 | UGAUUGAGCCGCGCCAAUAUCU | bna-miR171g |
| new miRNA 368 | UUUGGAUUGAAGGGAGCUCUA | bna-miR159 |
| new miRNA 369 | UGACAGAAGAGAGUGAGCAC | bna-miR156e |
| new miRNA 370 | UAGCCAAGGAUGACUUGCCUA | bna-miR169d |
| new miRNA 371 | UGGAGAGUAAGGACAUUCAGA | bna-miR6028 |
| new miRNA 372 | UUGAGCCGUGCCAAUAUCACG | bna-miR171d |
| new miRNA 373 | CAGCCAAGGAUGACUUGCCGA | bna-miR169b |
| new miRNA 374 | UCGGACCAGGCUUCAUCCCCC | bna-miR166f |
| new miRNA 375 | AUAGUACUAGUACUUGCAUGAUCA | bna-miR6036 |
| new miRNA 376 | UCAUUGAGUGCAGCGUUGAUGU | bna-miR397a |
| new miRNA 377 | UGAUUGAGCCGCGCCAAUAUC | bna-miR171f |
| new miRNA 378 | UGGAGUAGAAAAUGCAGUCGU | bna-miR6035 |
| new miRNA 379 | UAGCCAAGGAUGACUUGCCUA | bna-miR169f |
| new miRNA 380 | UGCCUGGCUCCCUGUAUGCCA | bna-miR160a |
| new miRNA 381 | CUGAAGUGUUUGGGGGAACUC | bna-miR395a |
| new miRNA 382 | GGAAUCUUGAUGAUGCUGCAU | bna-miR172b |
| new miRNA 383 | UAGCCAAGGAUGACUUGCCUGC | bna-miR169h |
| new miRNA 384 | UGCCAAAGGAGAUUUGCCCGG | bna-miR399a |
| new miRNA 385 | UGCCAAAGGAGAUUUGCCCGG | bna-miR399b |
| new miRNA 386 | CAGCCAAGGAUGACUUGCCGG | bna-miR169n |
| new miRNA 387 | UUGGCAUUCUGUCCACCUCC | bna-miR394b |
| new miRNA 388 | UCGGACCAGGCUUCAUUCCCC | bna-miR166a |
| new miRNA 389 | UGAAGCUGCCAGCAUGAUCUA | bna-miR167c |
| new miRNA 390 | CUGAAGUGUUUGGGGGGACUC | bna-miR395f |
| new miRNA 391 | UUCCACAGCUUUCUUGAACUU | bna-miR396a |
| new miRNA 392 | UUGAGCCGUGCCAAUAUCACG | bna-miR171b |
| new miRNA 393 | UCGAUAAACCUGUGCAUCCAG | bna-miR162a |
| new miRNA 394 | CUGAAGUGUUUGGGGGGACUC | bna-miR395d |
| new miRNA 395 | UGCCAAAGGAGAUUUGUCCGG | bna-miR399c |
| new miRNA 396 | UAGCCAAGGAUGACUUGCCUA | bna-miR169e |
| new miRNA 397 | GGAAUCUUGAUGAUGCUGCAU | bna-miR172c |
| new miRNA 398 | UAAUCUGCAUCCUGAGGUUUA | bna-miR2111b-5p |
| new miRNA 399 | UGCCUGGCUCCCUGUAUGCCA | bna-miR160d |
| new miRNA 400 | UUGGCAUUCUGUCCACCUCC | bna-miR394a |
| new miRNA 401 | GUCCUCGGGAUGCGGAUUACC | bna-miR2111a-3p |
| new miRNA 402 | UGACAGAAGAGAGUGAGCACA | bna-miR156a |
| new miRNA 403 | UAGCCAAGGAUGACUUGCCUGC | bna-miR169i |
| new miRNA 404 | UUGAGCCGUGCCAAUAUCACG | bna-miR171e |
| new miRNA 405 | UGAGCCAAAGAUGACUUGCCG | bna-miR169m |
| new miRNA 406 | UUAGAUUCACGCACAAACUCG | bna-miR403 |
| new miRNA 407 | UUGACAGAAGAUAGAGAGCAC | bna-miR156c |
| new miRNA 408 | UUGAGCCGUGCCAAUAUCACG | bna-miR171c |
| new miRNA 409 | AAGCUCAGGAGGGAUAGCGCC | bna-miR390c |
| new miRNA 410 | UCAAUACAUUGGACUACAUAU | bna-miR860 |
| new miRNA 411 | UGGGGUUGUGAUUUCAGGCUU | bna-miR6029 |
| new miRNA 412 | CUGAAGUGUUUGGGGGGACUC | bna-miR395e |
| new miRNA 413 | UGAACCAGAUAGAGUGGGACU | bna-miR6033 |
| new miRNA 414 | UCUGAUGUAUAUAGCUUUGGG | bna-miR6034 |
| new miRNA 415 | UAAUCUGCAUCCUGGGGUUUA | bna-miR2111c |
| new miRNA 416 | UGGAGAAGCAGGGCACGUGCG | bna-miR164c |
| new miRNA 417 | UAGCCAAGGAUGACUUGCCUA | bna-miR169c |
| new miRNA 418 | UAGCCAAGGAUGACUUGCCUGC | bna-miR169j |
| new miRNA 419 | UAAUCUGCAUCCUGAGGUUUA | bna-miR2111d |
| new miRNA 420 | UAAUCUGCAUCCUGAGGUUUA | bna-miR2111a-5p |
| new miRNA 421 | UGGAGAAGCAGGGCACGUGCG | bna-miR164d |
| new miRNA 422 | UUGACAGAAGAUAGAGAGCAC | bna-miR156b |
| new miRNA 423 | CUGAAGUGUUUGGGGGAACUC | bna-miR395c |
| new miRNA 424 | UCGCUUGGUGCAGGUCGGGAA | bna-miR168a |
| new miRNA 425 | AGAAUCUUGAUGAUGCUGCAG | bna-miR172d |
| new miRNA 426 | UGGAGCAUCAACAGAUCUCGG | bna-miR6032 |
| new miRNA 427 | AAGAGGUUCGGAGCGGUUUGAAGC | bna-miR6031 |
| new miRNA 428 | UGACAGAAGAGAGUGAGCAC | bna-miR156f |
| new miRNA 429 | UGAAGCUGCCAGCAUGAUCU | bna-miR167d |
| new miRNA 430 | UCGGACCAGGCUUCAUUCCCC | bna-miR166c |
| new miRNA 431 | GGAGUGUCAUGAGAACACGGA | bol-miR398a-5p |
| new miRNA 432 | AGAAUCUUGAUGAUGCUGCAU | bol-miR172b |
| new miRNA 433 | UUGAGCCGUGCCAAUAUCACG | bol-miR171a |
| new miRNA 434 | UAGACCAUUUGUGAGAAGGGA | bol-miR824 |
| new miRNA 435 | UGUGUUCUCAGGUCACCCCUU | bol-miR398a-3p |
| new miRNA 436 | AGAAUCUUGAUGAUGCUGCAU | bol-miR172a |
| new miRNA 437 | UUGACAGAAGAUAGAGAGCAC | bol-miR157a |
| new miRNA 438 | ACGUGAUAAGCCUCUGAAGAA | bra-miR5715 |
| new miRNA 439 | UGGAGAAGCAGGGCACGUGCA | bra-miR164a |
| new miRNA 440 | UGAAGCUGCCAGCAUGAUCUA | bra-miR167b |
| new miRNA 441 | GUUUGGAUUGUUUGCCUUGGC | bra-miR5717 |
| new miRNA 442 | AAUAUUAAUAUAAUUGGUGAG | bra-miR5712 |
| new miRNA 443 | UGAAGCUGCCAGCAUGAUCUA | bra-miR167d |
| new miRNA 444 | UUUGGAUUGAAGGGAGCUCUA | bra-miR159a |
| new miRNA 445 | AAAAAUGGAGUGAGAAAUGGA | bra-miR5721 |
| new miRNA 446 | UUGUGAUGAUAAUACGACUUC | bra-miR5719 |
| new miRNA 447 | UGAUUGAGCCGCGCCAAUAUC | bra-miR171e |
| new miRNA 448 | ACAGCCUAAACCAAUCGGAGC | bra-miR1140 |
| new miRNA 449 | AGGCUUAGAAGAACGUUUGUU | bra-miR5713 |
| new miRNA 450 | AGAAUCUUGAUGAUGCUGCAU | bra-miR172b-3p |
| new miRNA 451 | UGAAGCUGCCAGCAUGAUCUA | bra-miR167a |
| new miRNA 452 | AUUUGGCACAAUCUGAUCUGC | bra-miR5725 |
| new miRNA 453 | UUGAGCCGUGCCAAUAUCACG | bra-miR171b |
| new miRNA 454 | UUGAGCCGUGCCAAUAUCACG | bra-miR171a |
| new miRNA 455 | UAGACCAUUUGUGAGAAGGGA | bra-miR824 |
| new miRNA 456 | UACAUCUUCUCCGCGGAAGCUC | bra-miR1885b |
| new miRNA 457 | CAAAGGUUGCUUGAAUAAGGU | bra-miR5726 |
| new miRNA 458 | AGAAUCUUGAUGAUGCUGCAU | bra-miR172a |
| new miRNA 459 | GCAGCACCAUUAAGAUUCACA | bra-miR172b-5p |
| new miRNA 460 | AUCCUCGGGAUACGGAUUACC | bra-miR2111b-3p |
| new miRNA 461 | UUGUGAUUUGGUUGGAAUAUC | bra-miR5720 |
| new miRNA 462 | UCAGAACCAAACACAGAACAAG | bra-miR5718 |
| new miRNA 463 | UGCCUGGCUCCCUGUAUGCCA | bra-miR160a-5p |
| new miRNA 464 | UUGAGCCGUGCCAAUAUCACG | bra-miR171d |
| new miRNA 465 | UUGGAUAAUUGAAGAUAUAAA | bra-miR5716 |
| new miRNA 466 | GUCCUCGGGAUGCGGAUUACC | bra-miR2111a-3p |
| new miRNA 467 | AACCGCCGGUUUGAUAAUAGC | bra-miR5724 |
| new miRNA 468 | UAAUCUGCAUCCUGAGGUUUA | bra-miR2111b-5p |
| new miRNA 469 | UGAAGCUGCCAGCAUGAUCUA | bra-miR167c |
| new miRNA 470 | UGAAAUAGAGUCAUGUGGAACG | bra-miR5722 |
| new miRNA 471 | UUGACAGAAGAUAGAGAGCAC | bra-miR157a |
| new miRNA 472 | AUAAAUCCCAAGCAUCAUCCA | bra-miR5654b |
| new miRNA 473 | AAUGUGCUGCAAUAUCUCUGC | bra-miR5723 |
| new miRNA 474 | UUGAGCCGUGCCAAUAUCACG | bra-miR171c |
| new miRNA 475 | CAUCAAUGAAAGGUAUGAUUCC | bra-miR1885a |
| new miRNA 476 | GCGUAUGAGGAGCCAUGCAUA | bra-miR160a-3p |
| new miRNA 477 | AUAAAUCCCAAGCAUCAUCCA | bra-miR5654a |
| new miRNA 478 | UGUUUUGUGGGUUUCUACCGA | bra-miR5711 |
| new miRNA 479 | UAAUCUGCAUCCUGAGGUUUA | bra-miR2111a-5p |
| new miRNA 480 | AGACUCUACGACAUCAAGAAAC | bra-miR5714 |
| new miRNA 481 | UCGAUAAACCUCUGCAUCCAG | cpa-miR162a |
| new miRNA 482 | AAGCUCAGGAGGGAUAGCGCC | cme-miR390b |
| new miRNA 483 | UGAAUCUUGAUGAUGCCGCAC | cme-miR172f |
| new miRNA 484 | UGCCUGGCUCCCUGAAUGCCA | cme-miR160d |
| new miRNA 485 | UAGCCAAAGAUGACUUGCCUG | cme-miR169c |
| new miRNA 486 | UGCCAAAGGAGAGUUGCCCUU | cme-miR399d |
| new miRNA 487 | UAGCCAAAAAUGACUUGCCUG | cme-miR169b |
| new miRNA 488 | UGAAGCUGCCAGCAUGAUCUG | cme-miR167d |
| new miRNA 489 | UGACAGAAGAGAGUGAGCAC | cme-miR156d |
| new miRNA 490 | UGCCAAAGGAGAUUUGCCCCG | cme-miR399a |
| new miRNA 491 | UUGGACUGAAGGGAGCUCCU | cme-miR319c |
| new miRNA 492 | AGAAUCUUGAUGAUGCUGCAG | cme-miR172e |
| new miRNA 493 | UCGAUAAACCUCUGCAUCCAG | cme-miR162 |
| new miRNA 494 | UCGCUUGGUGCAGGUCGGGA | cme-miR168 |
| new miRNA 495 | UCGGACCAGGCUUCAUUCCUC | cme-miR166e |
| new miRNA 496 | AGUCAAAUCUAAACGAUCGUGUAU | cme-miR7129 |
| new miRNA 497 | CUGAAGUGUUUGGGGGAACUC | cme-miR395e |
| new miRNA 498 | UGCAUUUGCACCUGCACCUU | cme-miR530a |
| new miRNA 499 | UGGAGAAGCAGGGCACGUGCU | cme-miR164a |
| new miRNA 500 | UUGACAGAAGAUAGAGAGCAC | cme-miR156b |
| new miRNA 501 | CUGAAGUGUUUGGGGGAACUC | cme-miR395d |
| new miRNA 502 | AGAAUCUUGAUGAUGCUGCAU | cme-miR172b |
| new miRNA 503 | UCGGACCAGGCUUCAUUCCC | cme-miR166g |
| new miRNA 504 | UGCCAAAGGAGAGUUGCCCUU | cme-miR399e |
| new miRNA 505 | UCGGACCAGGCUUCAUUCCCC | cme-miR166c |
| new miRNA 506 | UAGCCAAAAAUGACUUGCCUGC | cme-miR169m |
| new miRNA 507 | UGACAGAAGAGAGUGAGCAC | cme-miR156i |
| new miRNA 508 | UCCAAAGGGAUCGCAUUGAUC | cme-miR393b |
| new miRNA 509 | UGCCAAAGGAGAGUUGCCCUA | cme-miR399b |
| new miRNA 510 | UUCCACGGCUUUCUUGAACUG | cme-miR396e |
| new miRNA 511 | GGAAUCUUGAUGAUGCUGCAG | cme-miR172a |
| new miRNA 512 | UCGGACCAGGCUUCAUUCUC | cme-miR166i |
| new miRNA 513 | UCUUGCUCAAAUGAGUAUUCCA | cme-miR828 |
| new miRNA 514 | UAGCCAAAGAUGACUUGCCUG | cme-miR169d |
| new miRNA 515 | UCAAGCUGCCAGCAUGAUCUA | cme-miR167e |
| new miRNA 516 | UGCCUGGCUCCCUGUAUGCCA | cme-miR160a |
| new miRNA 517 | UCGGACCAGGCUUCAUUCCCC | cme-miR166b |
| new miRNA 518 | AAGCUCAGGAGGGAUAGCGCC | cme-miR390d |
| new miRNA 519 | UGGAGAAGCAGGGCACGUGCA | cme-miR164d |
| new miRNA 520 | AAGCUCAGGAGGGAUAGCGCC | cme-miR390c |
| new miRNA 521 | UUGGACUGAAGGGAGCUCCC | cme-miR319b |
| new miRNA 522 | UGAGCCAAGAAUGACUUGCCGGC | cme-miR169t |
